# Associations of 24-h urinary excretion of heavy metals and trace elements with estimated glomerular filtration rate and chronic kidney disease

**DOI:** 10.1038/s41598-026-51034-8

**Published:** 2026-04-29

**Authors:** Sisi Xie, Aurélien Thomas, Belen Ponte, Daniel Ackermann, Menno Pruijm, Murielle Bochud, Pedro Marques-Vidal

**Affiliations:** 1 Department of Medicine, Internal Medicine, Lausanne University Hospital (CHUV) and University of Lausanne, Office Bh10-642, Rue du Bugnon 46, 1011 Lausanne, Switzerland; 2 Faculty Unit of Toxicology, University Center of Legal Medicine Lausanne-Geneva, Lausanne University Hospital and University of Lausanne, Lausanne, Switzerland; 3 Unit of Forensic Toxicology and Chemistry, CURML, Lausanne and Geneva University Hospitals, Lausanne, Switzerland; 4 Division of Nephrology and Hypertension, Department of Medicine, University Hospital of Geneva (HUG), Geneva, Switzerland; 5 Department of Nephrology and Hypertension, Inselspital, Bern University Hospital and University of Bern, Bern, Switzerland; 6 Service of Nephrology and Hypertension, Lausanne University Hospital (CHUV) and University of Lausanne, Lausanne, Switzerland; 7 Department of Epidemiology and Health Systems, Unisanté, Lausanne, Switzerland

**Keywords:** Chronic kidney disease, Metals, heavy, Trace elements, Glomerular filtration rate, Urinary excretion, Biomarkers, Diseases, Nephrology, Risk factors

## Abstract

Chronic kidney disease (CKD) is a growing global health concern. Although certain elements have been implicated in CKD, evidence based on 24-h urinary excretion and kidney outcomes remains limited. Cross-sectional data from the SKIPOGH cohort (2008–2013) were used to assess 24-h urinary element excretion, estimated glomerular filtration rate (eGFR), and CKD. Associations were evaluated using multivariable regression, nonlinear modeling, mixture analysis, and fractional excretion. Among 923 participants (mean age 47.8 years, 52.0% female), higher 24-h urinary excretion of vanadium, chromium, cobalt, palladium, silver, cadmium, and mercury was positively associated with eGFR in multivariable analyses. Fractional excretion analyses showed associations consistent with differences in kidney handling. In addition, CKD was present in 67 participants (7.3%). Higher 24-h urinary excretion of copper and zinc was associated with increased odds of CKD, whereas urinary excretion of selenium, palladium, cadmium, and mercury was inversely associated with CKD, including after adjustment for plasma concentrations. After correction for multiple testing, associations for vanadium, chromium, and palladium with eGFR, and for copper and mercury with CKD, remained statistically significant. Several 24-h urinary element excretion measures were statistically associated with kidney function. Longitudinal studies are needed to clarify temporal relationships and underlying mechanisms.

**Supplementary Information:**

The online version contains supplementary material available at 10.1038/s41598-026-51034-8.

## Introduction

Chronic kidney disease (CKD) affects approximately 10% of the adult population and contributes to increased risks of cardiovascular events, mortality, and healthcare costs^1^. While hypertension and diabetes are well-established risk factors, environmental exposures, including heavy metals and trace elements, have been increasingly implicated in CKD development and progression. Among various environmental pollutants, elements, including potentially toxic elements and essential trace elements, warrant particular attention because of their environmental persistence, bioaccumulative properties, and widespread human exposure. Unlike many organic pollutants, elements are non-biodegradable and may accumulate in the human body, and the kidney, as the main organ responsible for filtration and excretion, is particularly vulnerable to element-related toxicity.

Exposure to elements, such as cadmium (Cd), lead (Pb), mercury (Hg), and arsenic (As), has been increasingly associated with CKD^2,3^. These elements have been shown to induce nephrotoxicity through mechanisms such as oxidative stress, mitochondrial dysfunction, inflammation, and fibrosis^4,5^. In contrast, elements like selenium (Se), copper (Cu), and zinc (Zn) are essential micronutrients^6–8^, making the interpretation of their kidney effects more complex. Although required for physiological functions, both deficiency and excess of essential trace elements may adversely affect kidney function. Moreover, combined exposures may act synergistically or antagonistically, requiring mixture-based analysis. Previous studies have mainly focused on plasma concentrations or spot urine samples. However, 24-h urinary excretion provides a more comprehensive assessment of daily element elimination, reduces variability related to urine dilution, and minimizes the need for creatinine adjustment. As such, it may better capture the integrated daily excretion and kidney handling of these elements, providing a complementary perspective to blood concentrations or spot urine measurements. Few studies have simultaneously evaluated individual and joint associations between 24-h urinary element excretion and kidney outcomes in the general population.

Therefore, this study used a multicenter population-based Swiss cohort to investigate the associations of the 24-h urinary excretion of 24 elements with kidney function, estimated by glomerular filtration rate (eGFR) or CKD presence. The term “elements” is used throughout the manuscript to collectively refer to the investigated heavy metals, metalloids, and trace elements for consistency.

## Materials and methods

### Research design and participants

The SKIPOGH study is a family-based cohort designed to assess the associations between environmental factors, kidney function, and hypertension in the general population^9^. Recruited individuals were aged 18–90 years and lived in the region of the three study centers involved (Bern, Geneva, and Lausanne). After an overnight fast, all participants underwent a study visit that included a questionnaire, a complete clinical examination, blood sampling, and 24-h urine collection. Samples were collected at baseline from 2008 to mid-2013 and stored at − 80 °C until analysis.

### Measurement of 24 elements

24-h urinary excretion of 24 elements was quantified using inductively coupled plasma mass spectrometry (ICP-MS) following a previously described protocol^10–12^. Details of the analytical procedure, isotopes, and detection limits are provided in Supplementary Methods and Supplementary Table S1.

Fractional excretion (FE) was estimated as the ratio of 24-h urinary excretion to the estimated daily filtered amount and expressed as a percentage. The daily filtered amount was approximated as plasma concentration × eGFR × 1440 min/day^13^. Because eGFR is an estimated and body-surface-area–indexed measure rather than directly measured GFR, FE values should be interpreted as indicators of kidney handling rather than exact measurements^14^.

Given the cross-sectional design, 24-h urinary element excretion was conceptualized as a composite measure reflecting both systemic exposure and kidney handling, rather than a pure exposure marker.

### Estimation of kidney function

Kidney function was assessed based on CKD status, defined using eGFR and urinary albumin-to-creatinine ratio (UACR). Serum creatinine levels were measured using the Jaffe method, and eGFR was calculated from serum creatinine according to the Chronic Kidney Disease Epidemiology Collaboration 2021 (CKD-EPI-2021) formula^14^. CKD was defined according to KDIGO criteria as an eGFR < 60 mL/min/1.73 m^2^ of body surface area or a UACR ≥ 3 mg/mmol^1^. UACR was available in 909 participants. For the 14 individuals with missing UACR values, CKD classification was based solely on the eGFR criterion (< 60 mL/min/1.73 m^2^). UACR was categorized as A1 (lower than 3 mg/mmol), A2 (between 3–30 mg/mmol), and A3 (higher than 30 mg/mmol). Kidney function was analyzed both as a continuous outcome (eGFR) and as CKD to evaluate associations across the full spectrum of kidney function and clinically defined impairment.

### Covariates

Covariates were selected a priori based on prior epidemiological evidence and biological plausibility as potential confounders of the association between element exposure and kidney function. Similar covariate adjustment approaches have been used in previous population-based studies^15,16^. A directed acyclic graph was used to guide covariate selection (Supplementary Figure S1). In addition, a conceptual framework was constructed to clarify the dual role of urinary element excretion as reflecting both systemic exposure and kidney handling processes in this cross-sectional setting (Supplementary Figure S2).

We selected covariates among available demographic, clinical and laboratory data: age, sex, education, marital status, alcohol consumption, smoking, hypertension, diabetes, obesity, C-reactive protein, 25-hydroxyvitamin D, physical activity, and study center. Detailed variable definitions and measurement methods are provided in the Supplementary Methods.

### Exclusion criteria

For this study, all participants with available baseline data were considered eligible. We then excluded participants with (1) missing element data, (2) missing eGFR data, and (3) missing at least one of the covariates defined previously. Given that the majority of missing data concerned the 24-h urinary element excretion variables, and that these were missing as complete exposure panels rather than selectively across elements, complete-case analysis was performed.

### Statistical analysis

Statistical analyses were conducted using Stata v.18 (Stata Corp, College Station, TX, USA) and R version 4.4.2 (R Foundation for Statistical Computing, Vienna, Austria). Descriptive results were expressed as the number of participants (percentage) for categorical variables and as average ± standard deviation or median [interquartile range] for continuous variables. Between-group comparisons were conducted using chi-square for categorical variables and Student’s t-test or Kruskal–Wallis test for continuous variables.

In this study, 24-h urinary excretion of the 24 elements (in ng/day) was natural log-transformed to approximate normal distributions. Plasma concentrations were also natural log-transformed before inclusion in the models. To facilitate interpretation, continuous associations were expressed per doubling of 24-h urinary excretion by rescaling the natural log-transformed variables to log2 units (ln(X)/ln(2)). Pearson correlation coefficients were calculated to assess pairwise linear associations among the 24 elements. Associations between urinary element excretion and CKD were assessed using multivariable logistic regression, while linear regression was applied to evaluate associations with eGFR. Each element was analyzed in a separate multivariable regression model, adjusting for the predefined covariates, rather than being entered simultaneously into a single model. Elements were analyzed both as continuous variables and as quartiles to assess dose–response patterns. Trends across quartiles were evaluated using median values.

Restricted cubic spline (RCS) models were used to assess potential non-linear associations, with knot placement specified in Supplementary Methods. Mixture analyses were performed using weighted quantile sum (WQS) regression with least absolute shrinkage and selection operator (LASSO) selection, with model specification described in Supplementary Methods. To evaluate the robustness of the LASSO–WQS approach, sensitivity analyses were conducted using two alternative mixture modeling strategies: (1) WQS models including all 24 elements without LASSO pre-selection, and (2) quantile g-computation (qgcomp). Both models were adjusted for the same covariates as in the primary analyses.

All models were adjusted for covariates as previously defined. To assess potential overadjustment, minimally adjusted models including only age, sex, and study center were additionally performed as a sensitivity analysis. Standard errors were clustered by family code to account for familial correlations. Given the limited number of CKD cases, we assessed model stability by reporting the events-per-variable (EPV) for fully adjusted logistic regression models. As a sensitivity analysis, Firth penalized logistic regression was additionally performed for CKD to reduce potential small-sample bias under low EPV conditions.

As an additional analysis, models were adjusted for plasma element concentrations to account for systemic exposure levels and to distinguish exposure-related effects from kidney handling mechanisms. Moreover, FE of elements was analyzed as a complementary metric to estimate kidney handling relative to the filtered load, thereby providing additional insight beyond absolute urinary excretion. FE was evaluated in relation to CKD using logistic regression and to eGFR using linear regression models.

Statistical significance was defined as a two-sided test with p < 0.05. To account for multiple comparisons across the 24 elements, *P* values from the primary continuous models were adjusted using the Benjamini–Hochberg false discovery rate (FDR) procedure separately for each outcome (eGFR and CKD). FDR-adjusted* P* values (q-values) < 0.05 were considered statistically significant.

## Results

### Selection of participants

As shown in Supplementary Figure S3, of the 1,129 participants in the SKIPOGH study, 206 individuals were excluded due to missing data on 24-h urinary element excretion (n = 183), eGFR (n = 6), or relevant covariates (n = 17), resulting in 923 participants included in the final analysis. Supplementary Table S2 shows that included and excluded participants did not differ in baseline characteristics. Among the included participants, 52.0% were women, and 67 individuals (7.3%) had CKD. As shown in Table 1, participants with CKD were significantly older than those without CKD and had a higher prevalence of hypertension and diabetes. Participants with CKD had lower educational attainment and lower serum 25-hydroxyvitamin D concentrations.

**Table 1:** Characteristics of participants by kidney status. SKIPOGH study, Lausanne, Switzerland.

Variables	All participants (N = 923)	Non-CKD (N = 856)	CKD (N = 67)	*P*-value
Age, years	47.8 ± 17.6	46.8 ± 17.1	60.9 ± 18.7	**< 0.001**
Female sex, %	480 (52.0)	439 (51.3)	41 (61.2)	0.118
Education level, %				**0.002**
High	324 (35.7)	310 (36.8)	14 (21.5)	
Middle	415 (45.8)	386 (45.9)	29 (44.6)	
Low	168 (18.5)	146 (17.3)	22 (33.9)	
Marital status, %				0.441
Living alone	290 (31.5)	272 (31.9)	18 (27.3)	
Living in couple	630 (68.5)	582 (68.1)	48 (72.7)	
Smoking status, %				0.197
Never	414 (45.1)	383 (45.0)	31 (47.0)	
Former	286 (31.2)	261 (30.7)	25 (37.9)	
Current	217 (23.7)	207 (24.3)	10 (15.1)	
Alcohol consumption (%)				0.137
None	340 (36.8)	317 (37.0)	23 (34.3)	
1–13/week	404 (43.8)	380 (44.4)	24 (35.8)	
14–27/week	99 (10.7)	87 (10.2)	12 (17.9)	
28 + /week	80 (8.7)	72 (8.4)	8 (12.0)	
BMI group, %				0.154
Non-obese	797 (86.3)	743 (86.8)	54 (80.6)	
Obese	126 (13.7)	113 (13.2)	13 (19.4)	
Hypertension, %	293 (31.9)	251 (29.4)	42 (63.6)	**< 0.001**
Diabetes, %	43 (4.7)	35 (4.1)	8 (11.9)	**0.004**
Blood creatinine, µmol/L	73.6 ± 14.2	72.6 ± 12.2	87.0 ± 26.0	**< 0.001**
C-reactive protein (mg/L)	1.5[1.0–2.6]	1.5[1.0–2.9]	1.3[1.0–3.0]	0.479
Vitamin D (nmol/L)	92.5 ± 33.5	93.6 ± 32.9	79.3 ± 38.1	**< 0.001**
UACR categories				**< 0.001**
A1	866 (93.8)	843 (98.5)	23 (34.3)	
A2	39 (4.2)	0 (0)	39 (58.2)	
A3	4 (0.4)	0 (0)	4 (6.0)	
Missing	14(1.5)	13(1.5)	1(1.5)	
Urinary albumin (mg/L)	4.0[1.5–8.0]	4.0[1.5–7.0]	21.5[8.0–65.0]	**NR**
eGFR (mL/min/1.73 m^2^)	96.1 ± 17.9	97.8 ± 16.1	75.2 ± 25.2	**NR**

Results are expressed as the number of participants (column percentage) for categorical variables and as average ± standard deviation or median and [interquartile range] for continuous variables. Between-group comparisons were performed using chi-square for categorical variables and Student’s t-test or Kruskal–Wallis test for continuous variables. Abbreviations: CKD, chronic kidney disease; BMI, body mass index; eGFR, estimated glomerular filtration rate; UACR: urinary albumin-to-creatinine ratio; NR, not relevant.

Bold values indicate statistical significance (*P* < 0.05).

### Associations between individual elements of urinary excretion and eGFR

Associations between 24-h urinary excretion of elements and eGFR were examined using multivariable-adjusted linear regression (Table 2). Higher 24-h urinary excretion of V (β per doubling = 2.96, 95% CI: 1.41, 4.52), Cr (β = 2.19, 95% CI: 0.90, 3.48), Co (β = 0.70, 95% CI: 0.05, 1.35), Pd (β = 1.88, 95% CI: 0.87, 2.90), Ag (β = 0.64, 95% CI: 0.03, 1.25), Cd (β = 1.45, 95% CI: 0.14, 2.75), and Hg (β = 0.81, 95% CI: 0.08, 1.53) was associated with higher eGFR. After FDR correction, only V, Cr, and Pd remained statistically significant, whereas other associations did not retain significance. In minimally adjusted models that included only age, sex, and study center, the effect estimates were similar in direction and magnitude to those from the fully adjusted models (Supplementary Table S3).

**Table 2:** Associations of 24-h urinary excretion of individual elements (per doubling) with eGFR. SKIPOGH study, Lausanne, Switzerland.

Elements	Continuous			Quartiles				
	Coefficient (95% CI) per doubling	*P* value	FDR-q	Q1	Q2 Coefficient (95% CI)	Q3 Coefficient (95% CI)	Q4 Coefficient (95% CI)	*P* for trend
Li	0.09 (− 0.72, 0.90)	0.825	0.902	1 (ref.)	**− 2.70 (− 4.99, − 0.41)**	− 1.24 (− 3.61, 1.14)	− 0.02 (− 2.50, 2.47)	0.724
Be	0.53 (− 0.29, 1.36)	0.203	0.504	1 (ref.)	− 0.53 (− 2.93, 1.87)	0.09 (− 2.19, 2.38)	1.70 (− 0.71, 4.12)	0.134
Al	0.07 (− 0.72, 0.86)	0.864	0.902	1 (ref.)	0.35 (− 2.17, 2.87)	2.33 (− 0.01, 4.67)	0.06 (− 2.48, 2.60)	0.605
V	2.96 (1.41, 4.52)	**< 0.001**	**0.008**	1 (ref.)	0.99 (− 1.32, 3.31)	1.92 (− 0.60, 4.44)	**3.11 (0.50, 5.73)**	**0.019**
Cr	2.19 (0.90, 3.48)	**0.001**	**0.008**	1 (ref.)	0.66 (− 1.71, 3.04)	1.94 (− 0.39, 4.27)	**3.05 (0.77, 5.34)**	**0.008**
Mn	0.20 (− 0.48, 0.89)	0.560	0.84	1 (ref.)	0.62 (− 1.79, 3.04)	2.01 (− 0.53, 4.55)	1.52 (− 0.96, 4.00)	0.134
Co	0.70 (0.05, 1.35)	**0.036**	0.137	1 (ref.)	0.54 (− 1.72, 2.80)	0.34 (− 1.89, 2.56)	1.06 (− 1.33, 3.45)	0.431
Ni	0.39 (− 0.43, 1.21)	0.350	0.65	1 (ref.)	− 0.03 (− 2.66, 2.60)	− 0.58 (− 3.18, 2.03)	1.91 (− 0.87, 4.69)	0.238
Cu	0.16 (− 1.61, 1.93)	0.858	0.902	1 (ref.)	1.90 (− 0.62, 4.42)	0.62 (− 2.11, 3.36)	0.73 (− 2.32, 3.78)	0.855
Zn	− 0.19 (− 1.29, 0.92)	0.741	0.902	1 (ref.)	0.34 (− 1.91, 2.58)	− 0.98 (− 3.39, 1.42)	− 0.73 (− 3.50, 2.05)	0.428
As	− 0.06 (− 0.55, 0.42)	0.792	0.902	1 (ref.)	0.03 (− 2.21, 2.27)	0.65 (− 1.78, 3.08)	− 1.00 (− 3.59, 1.59)	0.563
Se	0.29 (− 1.38, 1.95)	0.735	0.902	1 (ref.)	− 0.11 (− 2.56, 2.33)	− 0.31 (− 2.70, 2.08)	0.29 (− 2.34, 2.93)	0.870
Mo	0.68 (− 0.38, 1.74)	0.210	0.504	1 (ref.)	− 0.24 (− 2.91, 2.43)	0.45 (− 2.14, 3.03)	0.66 (− 1.80, 3.12)	0.506
Pd	1.88 (0.87, 2.90)	**< 0.001**	**0.008**	1 (ref.)	2.07 (− 0.45, 4.59)	1.48 (− 0.83, 3.79)	**4.45 (1.92, 6.98)**	**0.002**
Ag	0.64 (0.03, 1.25)	**0.040**	0.137	1 (ref.)	1.32 (− 1.40, 4.04)	1.59 (− 0.92, 4.11)	**2.99 (0.44, 5.54)**	**0.024**
Cd	1.45 (0.14, 2.75)	**0.030**	0.137	1 (ref.)	− 0.89 (− 3.31, 1.52)	1.20 (− 1.47, 3.86)	2.55 (− 0.19, 5.30)	**0.033**
Sn	− 0.38 (− 1.18, 0.42)	0.352	0.65	1 (ref.)	− 0.91 (− 3.19, 1.37)	− 1.66 (− 4.00, 0.68)	− 0.62 (− 3.04, 1.80)	0.512
Sb	− 0.42 (− 1.18, 0.34)	0.281	0.613	1 (ref.)	− 0.16 (− 2.59, 2.27)	0.27 (− 2.40, 2.95)	− 0.71 (− 3.87, 2.44)	0.745
I	− 0.06 (− 1.30, 1.18)	0.921	0.921	1 (ref.)	− 1.25 (− 3.47, 0.97)	0.94 (− 1.36, 3.24)	0.57 (− 1.61, 2.74)	0.265
Pt	0.21 (− 0.67, 1.09)	0.635	0.896	1 (ref.)	0.56 (− 2.13, 3.26)	1.49 (− 0.86, 3.85)	− 0.32 (− 3.39, 2.75)	0.995
Hg	0.81 (0.08, 1.53)	**0.029**	0.137	1 (ref.)	0.14 (− 2.35, 2.63)	1.79 (− 0.57, 4.15)	1.73 (− 0.85, 4.32)	0.090
Tl	0.91 (− 0.38, 2.19)	0.165	0.495	1 (ref.)	0.34 (− 2.05, 2.74)	− 0.22 (− 2.51, 2.08)	2.37 (− 0.19, 4.93)	0.109
Pb	0.39 (− 0.53, 1.32)	0.407	0.698	1 (ref.)	0.14 (− 2.27, 2.55)	− 0.38 (− 2.86, 2.11)	1.99 (− 0.72, 4.71)	0.213
Bi	0.19 (− 0.40, 0.77)	0.528	0.840	1 (ref.)	− 1.06 (− 3.51, 1.39)	− 0.33 (− 3.04, 2.37)	0.60 (− 2.55, 3.75)	0.620

Results are presented as β coefficients and 95% confidence intervals (CI) from multivariable linear regression models adjusted for age, sex, education level, marital status, smoking status, alcohol consumption, obesity, hypertension, diabetes, vitamin D, C-reactive protein, physical activity, study center, and were clustered by family code. Continuous associations were assessed per doubling of 24-h urinary excretion using log2-transformed values (ln(X)/ln(2)). FDR *q*-values were computed using the Benjamini–Hochberg procedure across the 24 elements for each outcome. Quartile-based analyses were performed with the lowest quartile (Q1) as the reference group. *P* for trend was calculated by treating quartile categories as an ordinal variable in linear regression models.

Bold values indicate statistical significance (*P* < 0.05).

In quartile models, participants in the highest 24-h urinary excretion quartile (Q4) of V (β = 3.11, 95% CI: 0.50, 5.73), Cr (β = 3.05, 95% CI: 0.77, 5.34), Pd (β = 4.45, 95% CI: 1.92, 6.98), and Ag (β = 2.99, 95% CI: 0.44, 5.54) exhibited higher eGFR compared to those in Q1. Statistically significant trends across quartiles were observed for V, Cr, Pd, Ag, and Cd (all P for trend < 0.05).

### Associations between individual elements of urinary excretion and CKD

Similar association analyses were conducted with CKD as the outcome, using multivariable-adjusted logistic regression (Table 3). Higher 24-h urinary excretion of Cu (OR per doubling = 1.97, 95% CI: 1.30, 3.00) and Zn (OR = 1.73, 95% CI: 1.03, 2.65) was associated with increased odds of CKD. Conversely, higher 24-h urinary excretion of Se (OR = 0.61, 95% CI: 0.38, 0.97), Pd (OR = 0.60, 95% CI: 0.41, 0.87), Cd (OR = 0.68, 95% CI: 0.47, 0.97), and Hg (OR = 0.72, 95% CI: 0.58, 0.89) was associated with lower odds of CKD. After FDR correction, associations with Cu and Hg remained statistically significant, while other elements did not retain statistical significance. Effect estimates were similar in minimally adjusted models including only age, sex, and study center (Supplementary Table S3), with comparable directions and magnitudes for Cu, Zn, Se, Pd, and Hg.

**Table 3:** Associations of 24-h urinary excretion of individual elements (per doubling) with CKD. SKIPOGH study, Lausanne, Switzerland.

Elements	Continuous			Quartiles				
	OR (95% CI) per doubling	*P* value	FDR-q	Q1	Q2 OR (95% CI)	Q3 OR (95% CI)	Q4 OR (95% CI)	*P* for trend
Li	0.99 (0.77, 1.28)	0.949	0.949	1 (ref.)	0.88 (0.40, 1.92)	1.24 (0.57, 2.71)	0.87 (0.38, 2.01)	0.840
Be	0.98 (0.73, 1.31)	0.876	0.949	1 (ref.)	1.35 (0.60, 3.02)	1.05 (0.47, 2.34)	0.81 (0.33, 2.01)	0.572
Al	0.95 (0.76, 1.19)	0.684	0.864	1 (ref.)	1.10 (0.46, 2.61)	0.62 (0.28, 1.39)	0.85 (0.35, 2.04)	0.487
V	0.87 (0.53, 1.41)	0.572	0.763	1 (ref.)	0.83 (0.36, 1.95)	0.87 (0.37, 2.08)	0.83 (0.32, 2.14)	0.705
Cr	0.79 (0.55, 1.13)	0.199	0.423	1 (ref.)	2.10 (0.99, 4.45)	0.95 (0.39, 2.34)	0.82 (0.32, 2.08)	0.419
Mn	1.14 (0.93, 1.40)	0.219	0.423	1 (ref.)	1.93 (0.71, 5.30)	1.55 (0.60, 4.00)	2.03 (0.79, 5.24)	0.184
Co	1.02 (0.83, 1.26)	0.847	0.949	1 (ref.)	1.34 (0.64, 2.83)	0.78 (0.32, 1.89)	1.23 (0.57, 2.66)	0.845
Ni	0.78 (0.60, 1.01)	0.063	0.216	1 (ref.)	0.48 (0.20, 1.15)	0.76 (0.39, 1.50)	0.42 (0.18, 1.01)	0.084
Cu	1.97 (1.30, 3.00)	**0.002**	**0.036**	1 (ref.)	2.23 (0.83, 5.97)	1.75 (0.60, 5.15)	**3.99 (1.34**, **11.90)**	**0.017**
Zn	1.73 (1.13, 2.65)	**0.011**	0.066	1 (ref.)	1.72 (0.62, 4.76)	1.30 (0.48, 3.53)	**3.60 (1.35**, **9.59)**	**0.019**
As	1.10 (0.94, 1.27)	0.228	0.423	1 (ref.)	0.77 (0.29, 2.05)	1.03 (0.41, 2.59)	1.40 (0.62, 3.17)	0.295
Se	0.61 (0.38, 0.97)	**0.037**	0.148	1 (ref.)	0.51 (0.22, 1.19)	0.40 (0.16, 1.00)	0.54 (0.24, 1.25)	0.107
Mo	0.82 (0.62, 1.08)	0.155	0.413	1 (ref.)	1.52 (0.77, 2.98)	0.96 (0.43, 2.15)	0.92 (0.41, 2.06)	0.743
Pd	0.60 (0.41, 0.87)	**0.007**	0.056	1 (ref.)	**0.40 (0.17**, **0.96)**	**0.45 (0.20**, **1.00)**	**0.30 (0.12**, **0.71)**	**0.008**
Ag	1.09 (0.90, 1.32)	0.387	0.663	1 (ref.)	1.53 (0.62, 3.79)	1.22 (0.48, 3.06)	1.31 (0.51, 3.36)	0.713
Cd	0.68 (0.47, 0.97)	**0.034**	0.148	1 (ref.)	1.15 (0.52, 2.53)	0.90 (0.41, 1.99)	0.42 (0.17, 1.01)	**0.020**
Sn	0.93 (0.72, 1.19)	0.545	0.763	1 (ref.)	0.58 (0.24, 1.41)	0.58 (0.26, 1.30)	0.64 (0.29, 1.38)	0.303
Sb	1.20 (0.89, 1.61)	0.229	0.423	1 (ref.)	1.31 (0.62, 2.75)	0.57 (0.22, 1.49)	1.23 (0.39, 3.91)	0.947
I	1.11 (0.86, 1.44)	0.433	0.693	1 (ref.)	1.19 (0.51, 2.78)	1.23 (0.55, 2.74)	0.90 (0.39, 2.06)	0.826
Pt	1.02 (0.74, 1.40)	0.921	0.949	1 (ref.)	1.31 (0.47, 3.64)	0.95 (0.35, 2.59)	1.11 (0.43, 2.88)	0.983
Hg	0.72 (0.58, 0.89)	**0.003**	**0.036**	1 (ref.)	1.02 (0.50, 2.08)	**0.42 (0.18**, **0.99)**	0.48 (0.21, 1.09)	**0.025**
Tl	0.86 (0.55, 1.35)	0.518	0.763	1 (ref.)	1.11 (0.50, 2.45)	0.79 (0.32, 1.96)	0.86 (0.35, 2.09)	0.617
Pb	0.99 (0.75, 1.30)	0.939	0.949	1 (ref.)	0.73 (0.28, 1.91)	0.87 (0.38, 1.98)	0.75 (0.33, 1.68)	0.615
Bi	1.14 (0.96, 1.34)	0.125	0.375	1 (ref.)	1.12 (0.44, 2.86)	1.68 (0.67, 4.25)	1.65 (0.60, 4.53)	0.265

Results are expressed as odds ratios (OR) with 95% confidence intervals (CI). Logistic regression models were adjusted for age, sex, education level, marital status, smoking, alcohol consumption, physical activity, obesity, hypertension, diabetes, vitamin D, and C-reactive protein, study center, and accounted for familial clustering using robust standard errors. The continuous model represents the association per doubling of 24-h urinary excretion using log2-transformed values (ln(X)/ln(2)). FDR *q*-values were computed using the Benjamini–Hochberg procedure across the 24 elements for each outcome. Quartile models compare each quartile (Q2–Q4) to the reference (Q1), and *p* for trend was calculated by modeling the median value of each quartile as a continuous variable.

Bold values indicate statistical significance (*P* < 0.05).

In quartile models, participants in the highest 24-h urinary excretion quartile (Q4) of Cu (OR = 3.99, 95% CI: 1.34, 11.90) and Zn (OR = 3.60, 95% CI: 1.35, 9.59) had greater odds of CKD compared to those in the lowest quartile (Q1). Q4 levels of Pd (OR = 0.30, 95% CI: 0.12, 0.71) were associated with lower odds. Statistically significant trends across quartiles were observed for Cu, Zn, Pd, Cd, and Hg (all *P* for trend < 0.05).

The fully adjusted CKD model included 20 estimated parameters, corresponding to an EPV of 3.35. To evaluate potential small-sample bias, we conducted Firth penalized logistic regression (Supplementary Table S4). Associations for Cu and Zn remained positive, and Pd and Hg remained inverse, with comparable magnitudes. Associations for Se and Cd were directionally consistent but attenuated and did not reach conventional significance in the Firth models.

### Nonlinear dose–response relationships between elements and eGFR

**Fig. 1:**
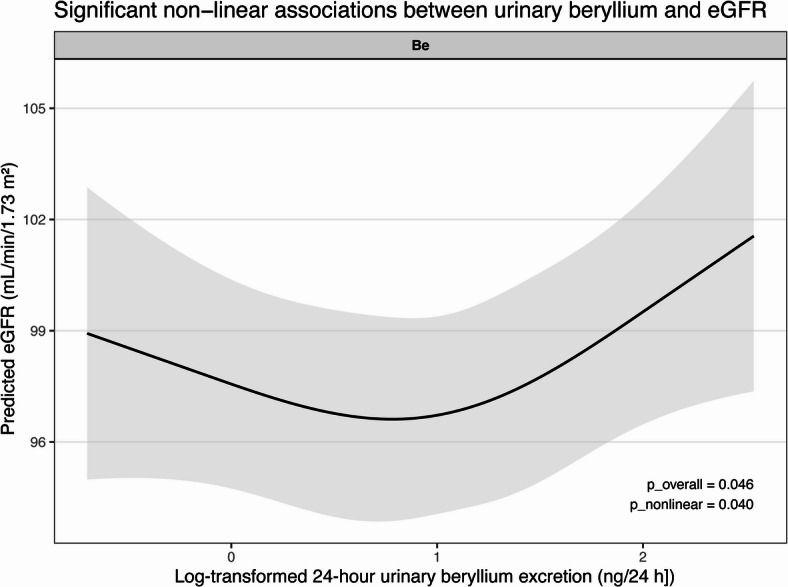
Non-linear association between log-transformed 24-h urinary excretion of beryllium and eGFR based on restricted cubic spline models. SKIPOGH study, Lausanne, Switzerland. Restricted cubic spline (RCS) models with four knots (5th, 35th, 65th, and 95th percentiles) were used to examine nonlinear associations between log-transformed 24-h urinary beryllium excretion (ng/24 h) and predicted eGFR (mL/min/1.73 m^2^). Solid lines represent adjusted predictions, and shaded areas indicate 95% confidence intervals. The 35th percentile was used as the reference value. P_overall tests the overall association, and P_nonlinear tests departure from linearity.

RCS models were used to examine the potential nonlinear associations between 24-h urinary excretion of 24 individual elements and eGFR. Among these, Be, Cd, Cr, Pd, and V exhibited significant overall associations with eGFR (*P* < 0.05) (Supplementary Figure S4). Beryllium (Be) demonstrated a statistically significant non-linear association with eGFR (*P* = 0.04), as shown in Fig. 1. The spline curve indicated a U-shaped pattern, with eGFR decreasing at lower Be levels and increasing at higher levels.

### Nonlinear dose–response relationships between elements and CKD

**Fig. 2:**
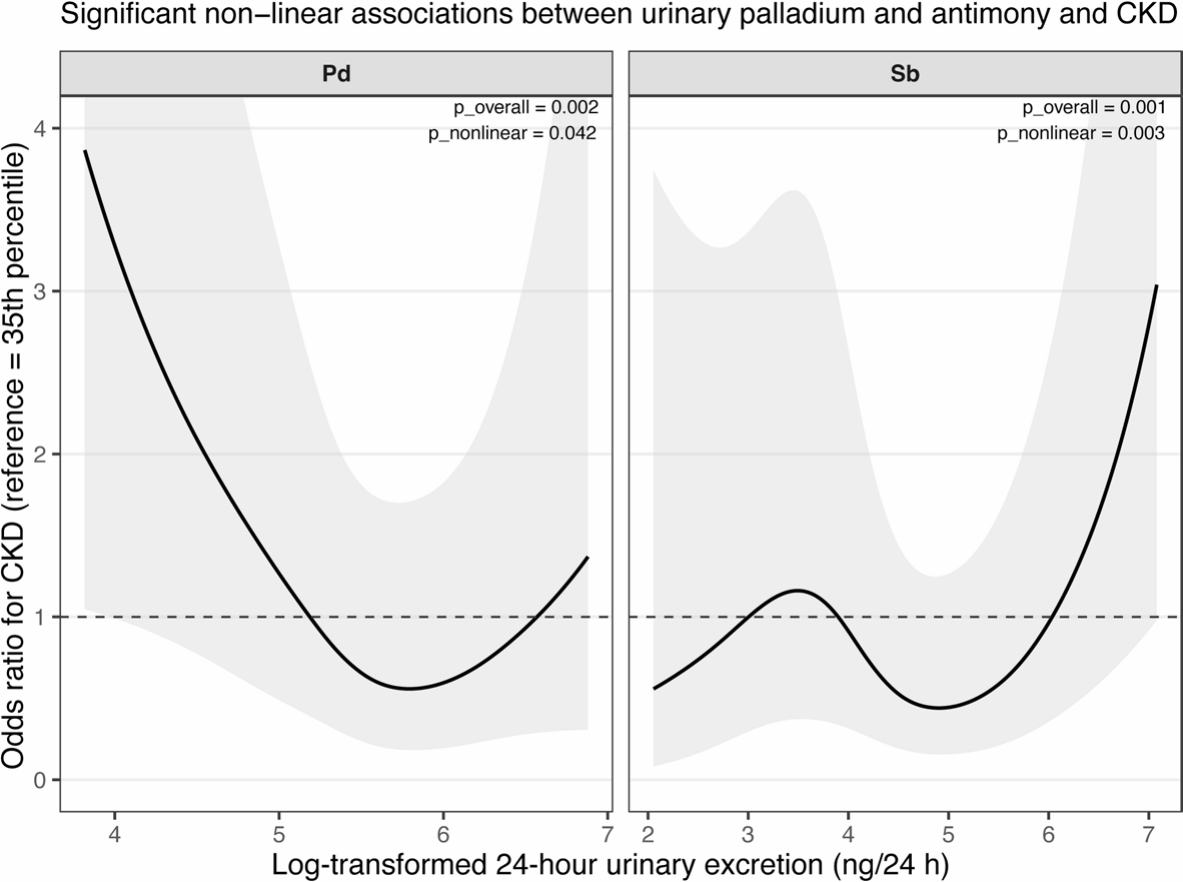
Non-linear associations between log-transformed 24-h urinary palladium and antimony excretion and CKD based on restricted cubic spline models. SKIPOGH study, Lausanne, Switzerland. Restricted cubic spline (RCS) models with four knots (5th, 35th, 65th, and 95th percentiles) were used to assess nonlinear associations between log-transformed 24-h urinary palladium and antimony excretion (ng/24 h) and odds of CKD. Solid lines represent multivariable-adjusted ORs, and shaded areas indicate 95% confidence intervals. The 35th percentile was used as the reference value. P_overall tests the overall association, and P_nonlinear tests departure from linearity.

RCS models were also used to examine the potential nonlinear associations between 24-h urinary excretion of 24 individual elements and CKD. Among these, Cu, Pd, and Sb exhibited significant overall associations with CKD (*P* < 0.05), while Zn showed a borderline association (*P* = 0.059) (Supplementary Figure S5). As shown in Fig. 2**,** both Pd and Sb demonstrated statistically significant non-linear associations with CKD (*P* = 0.042 and 0.003, respectively). The spline curve for Pd showed a U-shaped pattern, with lower odds of CKD at mid-range concentrations. In contrast, Sb showed a J-shaped pattern, with higher odds observed at higher concentrations.

### Associations of element mixtures with eGFR and CKD

To evaluate the joint effects of co-exposure to multiple elements, WQS regression analyses were conducted separately for eGFR and CKD outcomes. Before WQS modeling, LASSO regression was used to select subsets of relevant elements from the full set of 24 candidates.

**Fig. 3:**
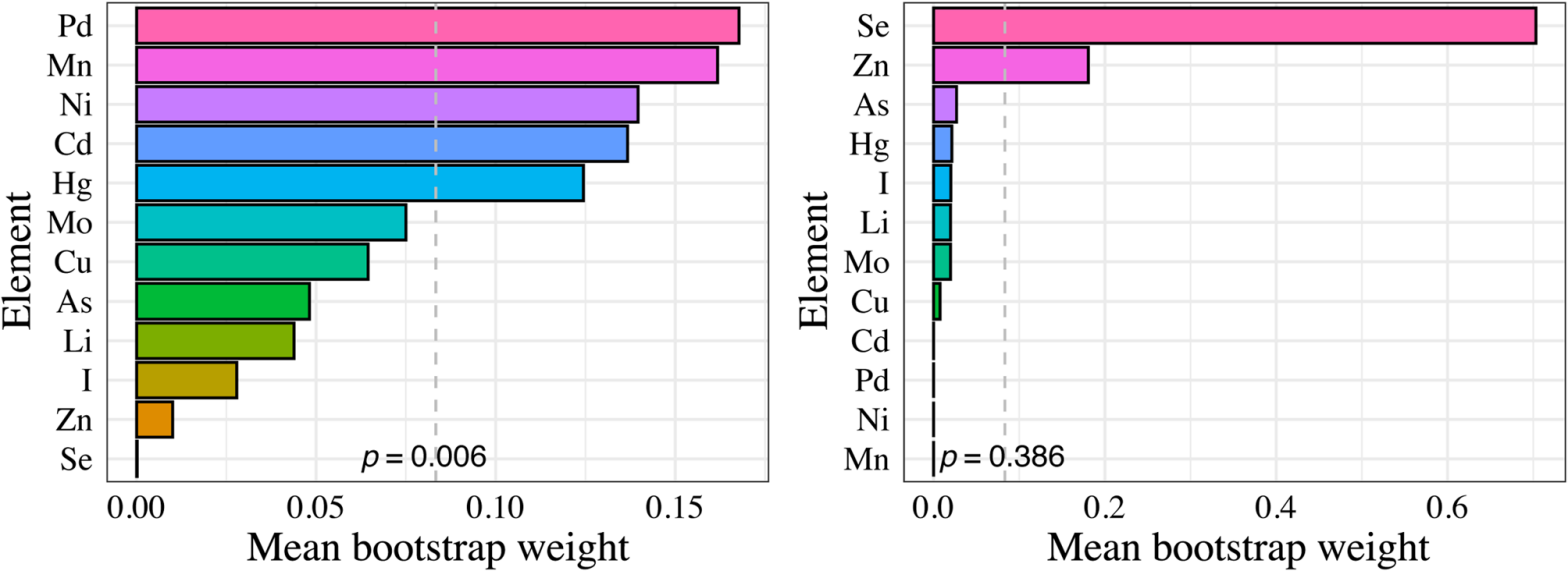
WQS regression results for the association between log-transformed 24-h urinary element excretion mixture and eGFR. SKIPOGH study, Lausanne, Switzerland. Weighted Quantile Sum (WQS) regression was performed using quartiles (q = 4) and 1,000 bootstrap samples with a 30% validation dataset. Separate models were fitted for positive (left panel) and negative (right panel) mixture directions. Bars represent mean bootstrap weights, with higher weights indicating stronger relative contributions to the mixture effect. The displayed p-values correspond to the overall association between the WQS index and eGFR in multivariable-adjusted linear regression models. Elements included in WQS models were pre-selected using LASSO regression from an initial set of 24 elements to reduce dimensionality and improve model stability.

For eGFR, 12 elements were retained by LASSO selection. A strong positive association was observed between the positively weighted element mixture and eGFR (*P* = 0.006) (Fig. 3, left panel), with Pd, Mn, Ni, Cd, and Hg contributing most prominently. The WQS model with negative weights did not reach significance (*P* = 0.386) (Fig. 3, right panel).

**Fig. 4:**
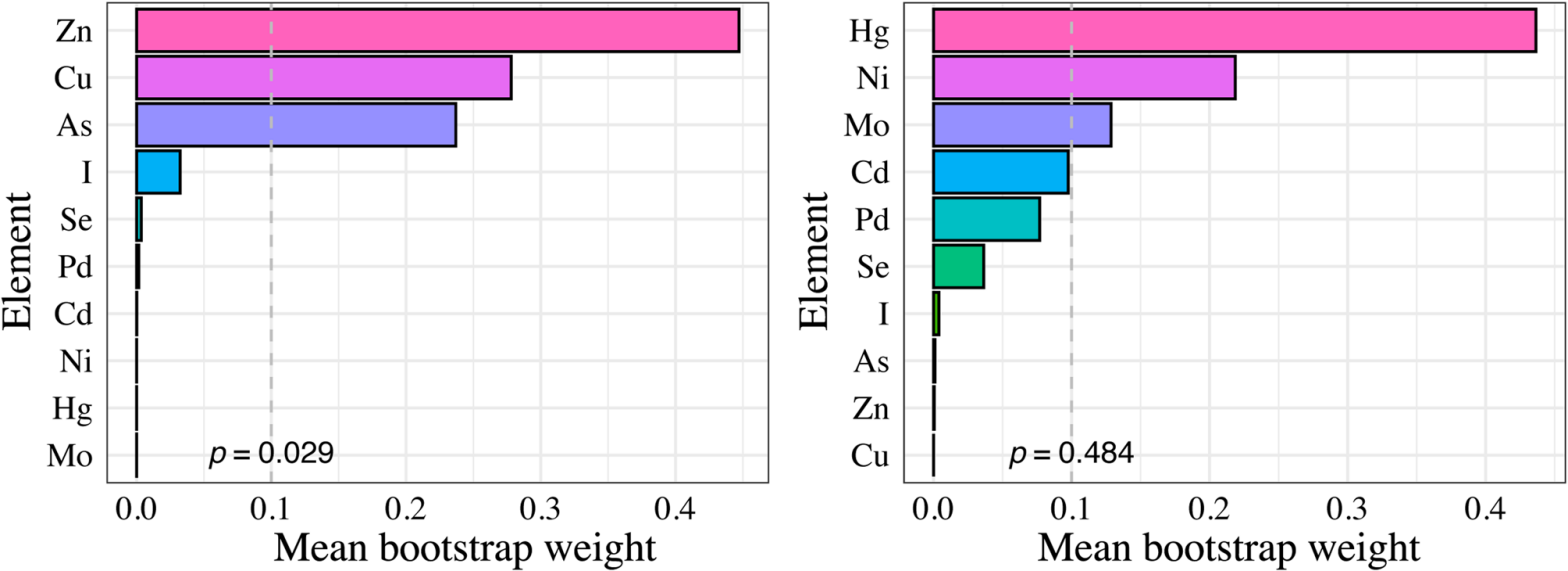
WQS regression results for the association between log-transformed 24-h urinary element excretion mixture and CKD. SKIPOGH study, Lausanne, Switzerland. Weighted Quantile Sum (WQS) regression was performed using quartiles (q = 4) and 1,000 bootstrap samples with a 30% validation dataset. Separate models were fitted for positive (left panel) and negative (right panel) mixture directions. Logistic regression models were used to estimate the association between the WQS index and CKD. Bars represent mean bootstrap weights, with higher weights indicating stronger relative contributions to the mixture effect. The displayed p-values correspond to the overall association between the WQS index and CKD in multivariable-adjusted logistic regression models. Elements included in the WQS models were pre-selected using LASSO regression from the initial set of 24 elements.

For CKD, 10 elements were retained by LASSO selection, and the WQS model identified a statistically significant positive association between the element mixture and CKD (*P* = 0.029) (Fig. 4, left panel). Zn, Cu, and As contributed the highest weights to the WQS index. In contrast, the WQS model with negative weights did not yield a statistically significant association (*P* = 0.484) (Fig. 4, right panel).

Sensitivity analyses using WQS models including all 24 elements and qgcomp models yielded broadly similar patterns for eGFR. For CKD, mixture associations were attenuated and no longer statistically significant (Supplementary Table S5).

Intercorrelations among the 24 elements are presented in Supplementary Figure S6 and were generally low to moderate.

### Additional analyses: adjusting for plasma element concentrations

We conducted additional analyses by further adjusting for plasma concentrations. These analyses evaluated whether associations between 24-h urinary excretion of elements and kidney outcomes were independent of plasma element levels. As shown in Supplementary Table S6, urinary and plasma levels of most elements showed weak to moderate correlations, although stronger positive correlations were observed for As and Co. Correlation coefficients ranged from − 0.105 to 0.749. Most correlations were positive, except for Cu and Sb.

As shown in Supplementary Table S7, after adjustment for plasma concentrations, the associations between urinary excretion of several elements and eGFR remained largely consistent. Positive associations with urinary levels of V, Cr, Co, Pd, and Hg persisted, and a new significant association emerged for Tl. In contrast, the associations with Ag and Cd were attenuated and lost statistical significance. Regarding CKD, higher urinary excretion of Cu and Zn continued to be significantly associated with increased odds of CKD, while Pd, Cd, and Hg remained inversely associated. Se was at the borderline of statistical significance (*P* = 0.055). Additionally, an inverse association with Mo emerged after adjusting for plasma element concentrations.

### Additional analyses: associations of fractional excretion with eGFR and CKD

To further evaluate the potential role of kidney handling of elements, we examined the associations between FE of individual elements and both eGFR and *CKD*.

For eGFR, higher FE of most elements was significantly associated with lower eGFR values. Al, V, Cr, Cu, Zn, Se, Mo, Cd, and Tl exhibited strong inverse associations (*P* < 0.001) (Supplementary Table S8).

For CKD, continuous models showed that higher FE of V, Cu, and Zn was significantly associated with increased odds of CKD (Supplementary Table S9). These associations were broadly consistent across quartiles, particularly for Q4 vs. Q1 comparisons.

## Discussion

### Main findings

In this cross-sectional population-based study, several elements were significantly associated with kidney function as measured by eGFR. Specifically, higher 24-h urinary excretion of V, Cr, Co, Pd, Ag, Cd, and Hg was associated with higher eGFR in multivariable-adjusted models. Many of these associations were also supported by results from WQS regression, RCS analyses, and further analyses adjusting for plasma concentrations. However, after correction for multiple testing using the FDR procedure, only V, Cr, and Pd remained statistically significant.

Because urinary excretion reflects both systemic exposure and kidney handling capacity, these measures should be interpreted as integrative markers rather than pure indicators of environmental exposure. Accordingly, the findings describe statistical associations and do not establish temporal directionality. Most elements showed inverse associations between FE and eGFR, which may reflect altered kidney handling during early functional changes rather than direct causal effects.

When CKD was evaluated as a binary outcome, higher 24-h urinary excretion of Cu and Zn was consistently associated with increased odds of CKD across multiple analytic approaches, including multivariable-adjusted logistic regression, dose–response modeling, WQS regression, and additional analyses adjusting for plasma concentrations. After FDR correction, only Cu (for positive associations) and Hg (for inverse associations with CKD) remained statistically significant. In contrast, Se, Pd, Cd, and Hg were associated with lower odds of CKD in the primary logistic regression models. These associations remained stable after further adjustment for plasma element concentrations. However, given the cross-sectional design, reverse causation cannot be excluded.

These elements originate from common environmental exposure pathways, including diet, tobacco smoke, seafood, and drinking water^17–19^. In Switzerland, maximum permissible concentrations of metals and metalloid contaminants in food are regulated under the Federal Ordinance on Contaminants, reflecting stringent food safety standards^20^. The 24-h urinary excretion levels observed in the SKIPOGH cohort were within the range reported in European population-based 24-h urine studies from Sweden and the United Kingdom^21,22^. Median excretion values for several elements (including Cd, Hg, Ni, Cu, Zn, and As) fell within published population intervals. Overall, these findings indicate background environmental exposure rather than unusual or extreme contamination, suggesting that the observed associations reflect variability within typical environmental exposure levels in the general population.

### Interpretation of associations between elements and eGFR

Several elements, including V, Cr, Co, Pd, Ag, Cd, and Hg, were positively associated with eGFR. These findings were directionally consistent with dose–response patterns, RCS analyses, positive weights in WQS regression, and plasma-adjusted models. One possible explanation is that higher urinary excretion may be consistent with preserved filtration and excretory function; however, alternative interpretations should be considered. Given the known nephrotoxic potential of some of these elements, the observed positive associations may also reflect changes in filtration dynamics, such as hyperfiltration, although the cross-sectional design does not allow determination of temporal directionality^2^.

In contrast, most elements showed negative associations between FE and eGFR, suggesting that individuals with lower eGFR excreted a greater fraction of filtered elements. This pattern may reflect impaired tubular reabsorption or altered kidney handling in early nephron dysfunction. Alternatively, increased FE may also represent an adaptive response aimed at enhancing the elimination of potentially toxic elements, analogous to regulatory mechanisms observed for phosphate handling in chronic kidney disease. Together, the positive associations for total excretion and inverse associations for FE suggest that urinary element levels mainly reflect filtration and clearance capacity rather than protective effects. Individuals with better kidney function may excrete larger total amounts due to higher filtered loads, whereas increased FE in those with lower eGFR likely reflects impaired tubular reabsorption or altered kidney handling. Because FE depends on plasma levels and kidney function, structural correlations with eGFR may occur. Therefore, FE findings should be interpreted cautiously and viewed as exploratory indicators of kidney handling rather than independent evidence of causal relationships.

### Interpretation of associations between elements and CKD: elements positively associated with CKD

Zn and Cu were consistently associated with increased odds across all analytical methods. As essential trace elements, both play critical roles in enzymatic and antioxidant defenses^8,23,24^.

For Zn, previous studies have reported that CKD patients exhibit lower circulating Zn levels accompanied by increased urinary Zn excretion, indicating impaired tubular reabsorption or compensatory kidney losses^25^. These alterations may be related to structural loss of functional tubules or dysfunction of specific Zn transporters in the proximal tubule, or proteinuria-mediated loss^26,27^. These findings are consistent with our observation that higher 24-h urinary excretion and FE of Zn were associated with increased odds of CKD, despite the physiological expectation that FE should decline in the context of low plasma Zn in CKD patients. The increased FE may indicate altered tubular handling rather than increased systemic exposure.

For Cu, consistent with our findings, several previous studies have reported a positive association between urinary copper levels and CKD^12,28,29^. Similarly, elevated urinary Cu excretion has been observed in kidney transplant recipients, particularly among individuals with proteinuria^30^. Protein-bound Cu may be increasingly filtered across a compromised glomerular barrier and insufficiently reabsorbed in damaged tubules. Cu overload may promote tubular injury through oxidative stress, lipid peroxidation, and inflammatory cytokine release^31–33^. Chelation studies in experimental settings suggest that Cu may participate in pathogenic processes rather than being solely a passive marker of damage^34^.

Overall, increased urinary Zn and Cu excretion may reflect dysregulated element homeostasis and tubular dysfunction. Whether these alterations contribute to CKD progression cannot be determined in this cross-sectional study.

### Interpretation of associations between elements and CKD: elements negatively associated with CKD

Se, Pd, Cd, and Hg showed inverse associations with CKD, contrasting with Cu and Zn. These findings are supported by previous studies reporting inverse associations between urinary Cd, Hg, and kidney function^2,35,36^. These associations remained after adjusting for plasma levels, suggesting they may reflect kidney-specific handling or early tubular dysfunction rather than systemic burden.

Se is a key component of antioxidant enzymes. Extensive research has demonstrated its beneficial impact on kidney function, with antioxidant activity identified as a principal underlying mechanism^6,37,38^. The inverse association with urinary Se may reflect a favorable antioxidant status or preserved tubular excretory function.

Pd has been less extensively studied in relation to kidney function. Notably, Pd exhibited a U-shaped dose–response relationship with CKD, suggesting a non-linear association across the exposure range, without implying a protective effect.

The inverse associations of Cd and Hg with CKD in our study should be interpreted with caution. Although nephrotoxic at high levels, low urinary Cd and Hg in CKD may reflect impaired excretion rather than protection. This may represent reverse causation, whereby declining filtration limits metal clearance. Additionally, early glomerular hyperfiltration from metals like Cd and Pb may transiently elevate urinary excretion in early injury^39^. As function declines, excretion falls, possibly contributing to inverse associations. Thus, reduced excretion likely indicates dysfunction, not protection.

These findings illustrate the complex interaction between exposure, kidney clearance, and oxidative stress. Further research is needed to clarify whether these elements act protectively, adaptively, or passively.

In this study, differences between the CKD and eGFR models may reflect their distinct definitions: CKD is a binary classification based on clinical thresholds, whereas eGFR, as a continuous variable, contains more information and may be more sensitive to early or subtle changes in kidney function. Together, these results highlight the multifaceted relationships between elements’ transport mechanisms and kidney physiology.

### Integration across analytical approaches and outcome definitions

Across analytical approaches, several elements (e.g., Cu, Zn, Pd, Cd, and Hg) showed directionally consistent associations in single-element regression and WQS analyses, with LASSO frequently identifying them as influential contributors. Some associations were not retained in mixture analyses, possibly due to shared exposure structures or model differences. RCS analyses further indicated nonlinear relationships for selected elements, although overall directions were generally aligned with primary regression findings.

Differences between continuous eGFR and categorical CKD outcomes likely reflect differences in statistical properties and clinical definition. Dichotomizing eGFR reduces information and may obscure subtle variation. Additionally, certain elements may be associated with variation in kidney function that does not cross the clinical CKD threshold, whereas others may be more strongly associated with clinically defined impairment. Overall, these approaches provide complementary rather than contradictory information.

### Strengths and limitations

This study has several strengths. First, 24-h urine collections provide a more comprehensive assessment of total daily urinary element excretion compared with spot urine samples, which may be influenced by short-term variability, diurnal fluctuation, and urine dilution^40–43^. Second, complementary analytical approaches addressed distinct methodological questions. Single-element models estimated exposure-specific associations, WQS evaluated mixture effects, LASSO supported variable selection in a multi-element context, and RCS explored potential nonlinear relationships, while FE analyses provided additional information on kidney handling.

However, limitations exist. First, the cross-sectional design precludes inference regarding temporal directionality. Because urinary element excretion is influenced by glomerular filtration and tubular transport, reverse causation cannot be excluded. Thus, differences in urinary excretion may reflect underlying kidney function or kidney handling processes rather than differences in systemic element levels. Second, although a complete-case analysis was applied, the included and excluded participants were comparable at baseline, suggesting limited evidence of major selection bias. However, residual bias cannot be entirely excluded. Third, FDR correction was applied to address multiple comparisons, yet the residual risk of false-positive findings cannot be entirely excluded. FE is mathematically dependent on plasma concentrations and kidney function measures, which may introduce structural correlations when examined in relation to eGFR or CKD. Thus, FE findings should be interpreted cautiously and regarded as exploratory indicators of kidney handling rather than independent evidence of causal effects. In addition, we did not assess long-term kidney outcomes or perform direct mechanistic studies. Finally, the number of CKD cases was relatively small, and most cases were moderate CKD. This limited statistical power may partly explain why mixture associations with CKD were attenuated in sensitivity analyses. Given the number of covariates included in the fully adjusted logistic regression models, the events-per-variable ratio was relatively low, which may limit model stability and the precision of effect estimates. Accordingly, we did not perform stratified analyses by CKD stage, as further subdivision would have resulted in unstable estimates. Therefore, associations with CKD should be interpreted with caution. Yet the population-based nature of the study increases the external validity of the findings.

## Conclusion

Our findings show several statistically significant associations between 24-h urinary element excretion and kidney function, some of which remained significant after adjustment for multiple comparisons. These results suggest that urinary element profiles may reflect differences in kidney physiology. However, given the cross-sectional design, longitudinal studies are required to clarify temporal relationships and potential clinical implications.

## Supplementary Information

Below is the link to the electronic supplementary material.

Supplementary Material 1

## Data Availability

SKIPOGH data can be made available upon request to SKIPOGH investigators as specified in the Maelström catalogue (Maelstrom Catalogue—Maelstrom Research). Requests for access to the datasets used and/or analysed during the current study can be addressed to the corresponding author and are subject to approval by the SKIPOGH study investigators in accordance with relevant governance procedures.
