# Supplementary material for: Associations of 24-h urinary excretion of heavy metals and trace elements with estimated glomerular filtration rate and chronic kidney disease
Source: Sci Rep. 2026 Apr 29;16:20025. doi: 10.1038/s41598-026-51034-8 (PMC13319113; doi:10.1038/s41598-026-51034-8)
Supplement: Supplementary file 1 — Supplementary Material 1 [file 41598_2026_51034_MOESM1_ESM.pdf]

Supplementary Information

Associations of 24-hour urinary excretion of heavy metals and trace elements with estimated glomerular filtration rate and chronic kidney disease

Sisi Xie[1], Aurélien Thomas[2,3], Belen Ponte[4], Daniel Ackermann[5], Menno Pruijm[6], Murielle Bochud[7] and Pedro Marques-Vidal[1]

1. Department of Medicine, Internal Medicine, Lausanne University Hospital (CHUV) and University of Lausanne, Lausanne, Switzerland.
2. Faculty Unit of Toxicology, University Center of Legal Medicine Lausanne-Geneva, Lausanne University Hospital and University of Lausanne, Lausanne, Switzerland
3. Unit of Forensic Toxicology and Chemistry, CURML, Lausanne and Geneva University Hospitals, Lausanne, Switzerland
4. Division of Nephrology and Hypertension, Department of Medicine, University Hospital of Geneva (HUG), Geneva, Switzerland.
5. Department of Nephrology and Hypertension, Inselspital, Bern University Hospital and University of Bern, Bern, Switzerland
6. Service of Nephrology and Hypertension, Lausanne University Hospital (CHUV) and University of Lausanne, Lausanne, Switzerland.
7. Department of Epidemiology and Health Systems, Unisanté, Lausanne, Switzerland

Corresponding author:
Pedro Marques-Vidal
Office Bh10-642, Centre Hospitalier Universitaire Vaudois (CHUV)
Rue du Bugnon 46, 1011 Lausanne, Switzerland
Phone: +41 (0)21 314 09 34
Email: Pedro-Manuel.Marques-Vidal@chuv.ch

Supplementary methods:

2.2. Measurement of 24 elements

24-hour urinary excretion of 24 elements was quantified using inductively coupled plasma mass spectrometry (ICP-MS) following a previously described protocol[10–12]. The 24 elements analyzed in this study were derived from the existing analytical panel measured within the SKIPOGH study. This panel was established to include elements of environmental relevance, encompassing toxic elements with prior evidence of nephrotoxicity and essential trace elements involved in kidney physiology and systemic homeostasis. The method involves acid dilution, internal standardization using rhodium and indium, multi-point calibration, and quality control with certified reference materials. Isotopes measured included ^{7}Li, ^{9}Be, ^{27}Al, ^{51}V, ^{53}Cr, ^{55}Mn, ^{59}Co, ^{60}Ni, ^{63}Cu, ^{66}Zn, ^{75}As, ^{82}Se, ^{95}Mo, ^{105}Pd, ^{107}Ag, ^{111}Cd, ^{118}Sn, ^{121}Sb, ^{127}I, ^{195}Pt, ^{201}Hg, ^{205}Tl, ^{208}Pb, and ^{209}Bi. **Supplementary Table S1** provides full element names, limit of detection (LOD) frequencies, and urinary concentration distributions. Values below the LOD were calculated as the corresponding LOD/2 at the concentration level (ng/mL).

2.4. Covariate definitions

We selected covariates among available demographic, clinical and laboratory data: age (years), sex (male/female), education (low/medium/high), marital status (living alone/living in couple), weekly alcohol consumption (None,1–13/week,14–27/week,28+/week), smoking (never/former/current), hypertension (yes/no), diabetes (yes/no), body mass index (BMI) (non-obese/obese), C-reactive protein (continuous), 25-hydroxyvitamin D3, physical activity (low/moderate/high), and study center (Lausanne, Geneva, and Bern).

Education and marital status were categorized based on questionnaire responses. Usual alcohol consumption during the week was self-reported and reported as the number of units (glasses of wine, bottles or cans of beer, and shots of spirits) per week. Smoking was self-reported and categorized as never, former (irrespective of the time since quitting smoking), and current. Physical activities were categorized as low, moderate, and high, based on weekly duration.

BMI was calculated from measured height and weight and categorized as non-obese (<30 kg/m^2), or obese (≥ 30 kg/m^2). Hypertension was defined by elevated office blood pressure (SBP ≥ 140 mm Hg or DBP ≥ 90 mm Hg) or use of antihypertensive medication. Diabetes was defined as fasting glucose ≥ 7.0 mmol/L or the use of antidiabetic medication. Plasma 25-hydroxyvitamin D3 was measured by a direct, competitive chemiluminescence immunoassay

on a LIAISON analyzer (DiaSorin S.p.A., Saluggia, Italy). The intra- and inter-assay coefficients of variation are 6.6% to 7.1% for plasma 25-hydroxyvitamin D3. CRP was measured using high sensitivity immunoturbidimetric assay.

2.6. Statistical analysis

Restricted cubic spline (RCS) models were used to assess potential non-linear associations between log-transformed 24-hour urinary element excretion and outcomes. Four knots were placed at the 5th, 35th, 65th, and 95th percentiles of the exposure distribution; the 35th percentile was used as the reference value. Weighted quantile sum (WQS) regression was conducted using quartiles (q = 4) and 1,000 bootstrap samples, with a 30% validation dataset to estimate mixture weights. Separate models were fitted for positive and negative directions by constraining the overall mixture effect to be positive or negative. WQS models estimate the overall directional association of the mixture with the outcome while deriving element-specific weights that sum to 1 and reflect relative contributions. Element selection was performed using the least absolute shrinkage and selection operator (LASSO) regression with penalized estimation to reduce dimensionality and improve model stability in the presence of multiple correlated exposures before inclusion in the WQS models. To evaluate the robustness of the LASSO–WQS approach, sensitivity analyses were conducted using two alternative mixture modeling strategies: (1) WQS models including all 24 elements without LASSO pre-selection, and (2) quantile g-computation (qgcomp). Both models were adjusted for the same covariates as in the primary analyses.

Supplementary Table S1: Abbreviations, full names, percentile distribution of 24-hour urinary excretion of elements, concentration, number, and percentage below the limit of detection (LOD). SKIPOGH study, Lausanne, Switzerland.

Abbrevi-ations	Full names	24-hour urinary excretion (ng)		24-hour urinary concentration (ng/ml)		
		Median	Interquartile Range	LOD	Number of observations below LOD (n and %)	
Li	Lithium	33692	23656 - 53544	0.437	0	0%
Be	Beryllium	2.92	1.89 - 4.31	0.002	506	54.82%
Al	Aluminum	5871	3612 - 9112	1.223	100	10.83%
V	Vanadium	752	567 - 962	0.024	0	0%
Cr	Chromium	1907	1416 - 2466	0.100	0	0%
Mn	Manganese	234	131 - 400	0.568	881	95.45%
Co	Cobalt	272	192 - 449	0.023	1	0.11%
Ni	Nickel	2183	1540 - 3100	0.044	0	0%
Cu	Copper	13108	9505 - 17232	2.712	43	4.66%
Zn	Zinc	325040	211372 - 501418	8.596	0	0%
As	Arsenic	17957	7856 - 43577	0.117	0	0%
Se	Selenium	27774	21454 - 35011	1.578	0	0%
Mo	Molybdenum	42000	27184 - 62178	0.054	0	0%
Pd	Palladium	225	150 - 318	0.009	1	0.11%
Ag	Silver	79.51	46.35 - 225	0.050	537	58.18%
Cd	Cadmium	295	198 - 417	0.042	15	1.63%
Sn	Tin	496	316 - 797	0.152	140	15.17%
Sb	Antimony	67.53	38.37 - 138	0.044	451	48.86%
I	Iodine	134974	103468 - 178600	2.497	0	0%
Pt	Platinum	46.29	30.68 - 68.35	0.020	239	25.89%
Hg	Mercury	393	211 - 661	0.094	122	13.22%
Tl	Thallium	239	178 - 332	0.003	0	0%
Pb	Lead	1188	708 - 1845	0.226	83	8.99%
Bi	Bismuth	14.78	6.52 - 45.09	0.010	458	49.62%

Supplementary Table S2: Characteristics of participants included vs. excluded from the analysis. SKIPOGH study, Lausanne, Switzerland.

Variables	Included (n=923)	Excluded (n=206)	*P-value*
Age, years	47.8 ± 17.6	45.6 ± 16.8	0.094
Female sex, %	480 (52.0)	109 (52.9)	0.813
Education level, %			0.559
High	324 (35.7)	78 (39.2)	
Middle	415 (45.8)	83 (41.7)	
Low	168 (18.5)	38 (19.1)	
Marital status, %			0.856
Living alone	290 (31.5)	65 (32.2)	
Living in couple	630 (68.5)	137 (67.8)	
Smoking status, %			0.489
Never	414 (45.1)	84 (42.2)	
Former	286 (31.2)	60 (30.2)	
Current	217 (23.7)	55 (27.6)	
Alcohol consumption (%)			0.150
None	340 (36.8)	67 (32.5)	
1-13/week	404 (43.8)	91 (44.2)	
14-27/week	99 (10.7)	20 (9.7)	
28+/week	80 (8.7)	28 (13.6)	
BMI group, %			0.139
Non-obese	797 (86.3)	184 (90.2)	
Obese	126 (13.7)	20 (9.8)	
Hypertension, %	293 (31.9)	53 (26.6)	0.149
Diabetes, %	43 (4.7)	8 (4.2)	0.751
Blood creatinine, µmol/L	73.6 ± 14.2	73.7 ± 14.1	0.962
C-reactive protein (mg/L)	1.5[1.0-2.6]	1.5[1.0-3.0]	0.180
Vitamin D (nmol/L)	92.5 ± 33.5	94.6 ± 34	0.464
Urinary albumin (mg/L)	4.0[1.5-8.0]	4.0[1.5-8.0]	1.000
Kidney function (%)			0.655
Normal	856 (92.7)	191 (92.7)	
CKD	67 (7.3)	13 (6.3)	
Missing	0(0)	2(1.0)	
eGFR (mL/min/1.73m^2)	96.1 ± 17.9	97.4 ± 18.1	0.383

Results are expressed as the number of participants (column percentage) for categorical variables and as average ± standard deviation or median and [interquartile range] for continuous variables. Between-group comparisons were performed using chi-square for categorical variables and Student's t-test or Kruskal-Wallis test for continuous variables. Abbreviations: CKD, chronic kidney disease; BMI, body mass index; eGFR, estimated glomerular filtration rate.

Supplementary Table S3: Minimally adjusted associations (age, sex, study center) between 24-hour urinary element excretion and kidney outcomes. SKIPOGH study, Lausanne, Switzerland.

Elements	eGFR		CKD	
	**Coefficient (95% CI) per doubling**	***P* value**	**OR (95% CI) per doubling**	***P* value**
Li	-0.04 (-0.80, 0.73)	0.928	1.00 (0.80, 1.26)	0.970
Be	0.50 (-0.27, 1.27)	0.205	1.05 (0.83, 1.35)	0.673
Al	0.02 (-0.67, 0.71)	0.949	0.99 (0.83, 1.17)	0.870
V	2.79 (1.36, 4.23)	**<0.001**	0.99 (0.65, 1.52)	0.971
Cr	2.05 (0.85, 3.24)	**0.001**	0.87 (0.63, 1.18)	0.371
Mn	0.03 (-0.62, 0.68)	0.936	1.15 (0.97, 1.35)	0.108
Co	0.57 (-0.03, 1.16)	0.060	1.03 (0.85, 1.26)	0.742
Ni	0.22 (-0.55, 0.98)	0.575	0.73 (0.57, 0.93)	**0.012**
Cu	-0.08 (-1.72, 1.57)	0.927	1.91 (1.33, 2.75)	**<0.001**
Zn	-0.04 (-1.04, 0.95)	0.934	1.56 (1.10, 2.21)	**0.012**
As	-0.13 (-0.57, 0.30)	0.552	1.05 (0.92, 1.21)	0.446
Se	-0.02 (-1.53, 1.49)	0.978	0.65 (0.43, 0.99)	**0.043**
Mo	0.73 (-0.25, 1.70)	0.142	0.87 (0.67, 1.12)	0.286
Pd	2.02 (1.06, 2.98)	**<0.001**	0.57 (0.40, 0.82)	**0.002**
Ag	0.66 (0.05, 1.26)	**0.034**	1.02 (0.86, 1.20)	0.855
Cd	1.28 (0.09, 2.47)	**0.035**	0.77 (0.55, 1.08)	0.136
Sn	-0.28 (-1.07, 0.50)	0.478	0.87 (0.67, 1.13)	0.302
Sb	-0.27 (-0.99, 0.44)	0.447	1.13 (0.85, 1.50)	0.395
I	0.25 (-0.84, 1.35)	0.647	1.05 (0.81, 1.36)	0.736
Pt	0.16 (-0.67, 0.99)	0.706	0.89 (0.67, 1.19)	0.441
Hg	0.79 (0.12, 1.46)	**0.020**	0.71 (0.60, 0.85)	**<0.001**
Tl	0.58 (-0.57, 1.72)	0.321	0.89 (0.60, 1.30)	0.540
Pb	0.46 (-0.35, 1.26)	0.264	0.99 (0.79, 1.25)	0.958
Bi	0.16 (-0.39, 0.70)	0.573	1.06 (0.90, 1.24)	0.501

Results are presented as regression coefficients (95% CI) for eGFR and odds ratios (95% CI) for CKD. Models were adjusted for age, sex, and study center. Estimates represent changes per doubling of 24-hour urinary element excretion (log_2-transformed scale). Robust standard errors clustered by family were used. These analyses were conducted to assess potential overadjustment in the fully adjusted models. P-values are not adjusted for multiple comparisons. Abbreviations: eGFR, estimated glomerular filtration rate; CKD, chronic kidney disease; CI, confidence interval.

Supplementary Table S4: Sensitivity analysis using Firth penalized logistic regression for CKD. SKIPOGH study, Lausanne, Switzerland.

**Elements**	**OR (95% CI) per doubling**	***P* value**
Li	1.00 (0.78,1.29)	0.978
Be	0.98 (0.73,1.32)	0.888
Al	0.96 (0.73,1.26)	0.773
V	0.87 (0.53,1.43)	0.577
Cr	0.80 (0.52,1.21)	0.286
Mn	1.13 (0.91,1.41)	0.251
Co	1.03 (0.82,1.30)	0.784
Ni	0.80 (0.59,1.09)	0.160
Cu	1.89 (1.20,2.98)	**0.006**
Zn	1.67 (1.13,2.47)	**0.009**
As	1.09 (0.94,1.27)	0.256
Se	0.63 (0.37,1.05)	0.079
Mo	0.83 (0.59,1.16)	0.275
Pd	0.62 (0.45,0.84)	**0.003**
Ag	1.08 (0.90,1.31)	0.398
Cd	0.69 (0.48,1.01)	0.056
Sn	0.93 (0.72,1.21)	0.603
Sb	1.20 (0.98,1.46)	0.083
I	1.11 (0.86,1.44)	0.413
Pt	1.03 (0.76,1.40)	0.854
Hg	0.74 (0.58,0.94)	**0.013**
Tl	0.89 (0.57,1.37)	0.589
Pb	0.98 (0.71,1.35)	0.904
Bi	1.13 (0.96,1.34)	0.138

Firth penalized logistic regression was applied for CKD to reduce small-sample bias in models with limited events. Models were adjusted for the same covariates as the fully adjusted models. Estimates are presented as odds ratios (95% CI) per doubling of 24-hour urinary element excretion (log_2-transformed scale). Abbreviations: CKD, chronic kidney disease; CI, confidence interval.

Supplementary Table S5: Sensitivity analyses of mixture models using alternative approaches (WQS including all 24 elements and quantile g-computation).

Outcome	Method	Elements included	Effect estimate (95% CI)	P value
eGFR	WQS (positive direction)	all 24 elements	2.85 (0.24, 5.46)	**0.034**
eGFR	Qgcomp	all 24 elements	2.52 (0.81, 4.24)	**0.004**
CKD	WQS (positive direction)	all 24 elements	1.92 (0.63, 5.81)	0.248
CKD	Qgcomp	all 24 elements	0.81 (0.40, 1.65)	0.559

Sensitivity analyses were conducted to evaluate the robustness of the primary LASSO→WQS approach. WQS models were re-fitted, including all 24 elements (without LASSO pre-selection) using quartiles (q = 4), 1,000 bootstrap samples, and a 70/30 training–validation split. Quantile g-computation (qgcomp) models were fitted using the same quantile specification (q = 4) with 1,000 bootstrap iterations. All models were adjusted for the same covariates as the fully adjusted main models. Effect estimates are reported as β coefficients for eGFR and odds ratios (ORs) for CKD, each with 95% confidence intervals. Both positive and negative directional WQS models were fitted; the direction corresponding to the primary analysis is reported.

Supplementary Table S6: Correlation between plasma concentrations and 24-hour urinary excretion levels of 24 elements. SKIPOGH study, Lausanne, Switzerland.

**Elements**	**Pearson r**	***P* value**
Li	0.394	**<0.001**
Be	0.027	0.434
Al	0.036	0.306
V	0.100	**0.004**
Cr	0.197	**<0.001**
Mn	-0.050	0.152
Co	0.724	**<0.001**
Ni	0.232	**<0.001**
Cu	-0.105	**0.003**
Zn	0.235	**<0.001**
As	0.749	**<0.001**
Se	0.336	**<0.001**
Mo	0.437	**<0.001**
Pd	0.146	**<0.001**
Ag	0.166	**<0.001**
Cd	0.205	**<0.001**
Sn	0.345	**<0.001**
Sb	-0.082	**0.018**
I	0.175	**<0.001**
Pt	0.111	**0.001**
Hg	0.587	**<0.001**
Tl	0.577	**<0.001**
Pb	0.230	**<0.001**
Bi	0.295	**<0.001**

Plasma element concentrations and 24-hour urinary excretion levels were log-transformed before analysis. Pearson correlation coefficients(r) were calculated, and P-values indicate the significance of the correlations.

Supplementary Table S7: Associations of 24-hour urinary excretion of 24 elements with eGFR and CKD, adjusted for plasma concentrations. SKIPOGH study, Lausanne, Switzerland.

**Elements**	**eGFR**		**CKD**	
	**Coefficient (95% CI)**	***P* value**	**OR (95% CI)**	***P* value**
Li	-0.19 (-1.57, 1.18)	0.782	0.79 (0.51, 1.24)	0.305
Be	0.82 (-0.40, 2.05)	0.187	0.94 (0.61, 1.46)	0.799
Al	-0.03 (-1.30, 1.24)	0.962	0.92 (0.62, 1.35)	0.663
V	3.04 (0.68, 5.39)	**0.012**	0.79 (0.36, 1.73)	0.561
Cr	2.20 (0.27, 4.14)	**0.026**	0.79 (0.41, 1.49)	0.464
Mn	0.25 (-0.81, 1.30)	0.644	1.08 (0.75, 1.57)	0.679
Co	2.36 (0.93, 3.78)	**0.001**	0.75 (0.42, 1.35)	0.340
Ni	0.39 (-0.79, 1.57)	0.512	0.68 (0.45, 1.02)	0.059
Cu	-0.24 (-2.97, 2.49)	0.864	2.38 (1.25, 4.54)	**0.009**
Zn	-0.43 (-2.15, 1.29)	0.622	2.26 (1.13, 4.52)	**0.021**
As	0.81 (-0.19, 1.82)	0.112	0.93 (0.61, 1.42)	0.736
Se	1.06 (-1.84, 3.96)	0.473	0.45 (0.20, 1.02)	0.055
Mo	1.10 (-0.73, 2.93)	0.237	0.49 (0.28, 0.86)	**0.013**
Pd	2.99 (1.39, 4.59)	**<0.001**	0.42 (0.23, 0.76)	**0.004**
Ag	0.62 (-0.33, 1.56)	0.199	1.05 (0.77, 1.44)	0.741
Cd	1.61 (-0.36, 3.58)	0.109	0.57 (0.33, 0.99)	**0.045**
Sn	-0.18 (-1.62, 1.26)	0.809	0.94 (0.64, 1.40)	0.778
Sb	-0.74 (-1.92, 0.44)	0.217	1.32 (0.84, 2.08)	0.231
I	0.08 (-1.59, 1.76)	0.921	0.75 (0.44, 1.30)	0.307
Pt	0.60 (-0.79, 1.99)	0.396	0.95 (0.58, 1.56)	0.854
Hg	2.31 (0.90, 3.71)	**0.001**	0.54 (0.36, 0.80)	**0.002**
Tl	2.95 (0.45, 5.45)	**0.021**	0.65 (0.27, 1.52)	0.317
Pb	0.57 (-0.83, 1.97)	0.426	0.86 (0.57, 1.28)	0.458
Bi	0.20 (-0.73, 1.13)	0.668	1.14 (0.86, 1.50)	0.359

Results are expressed as coefficients (95% CI) for eGFR from multivariable linear regression and odds ratios (95% CI) for CKD from multivariable logistic regression. Sensitivity analyses were performed by additionally adjusting for corresponding plasma trace element concentrations. 24-h urinary element excretion variables were log-transformed before analysis. Robust standard errors were used to account for clustering. Abbreviations: eGFR, estimated glomerular filtration rate; CKD, chronic kidney disease; CI, confidence interval.

Supplementary Table S8: Associations of fractional excretion of individual elements with eGFR. SKIPOGH study, Lausanne, Switzerland.

Elements	Continuous		Quartiles				
	**Coefficient (95% CI)**	***P* value**	**Q1**	**Q2 Coefficient (95% CI)**	**Q3 Coefficient (95% CI)**	**Q4 Coefficient (95% CI)**	***P* for trend**
Li	-1.31 (-2.13, -0.50)	**0.002**	1 (ref.)	0.59 (-2.09, 3.26)	**-3.24 (-5.70, -0.78)**	-2.18 (-4.78, 0.42)	**0.012**
Be	-1.03 (-1.61, -0.44)	**0.001**	1 (ref.)	-0.53 (-3.07, 2.01)	-1.73 (-4.07, 0.60)	**-4.31 (-6.73, -1.90)**	**<0.001**
Al	-1.47 (-2.29, -0.66)	**<0.001**	1 (ref.)	-1.54 (-3.87, 0.78)	**-4.64 (-7.41, -1.87)**	**-4.24 (-7.10, -1.38)**	**0.001**
V	-6.32 (-8.63, -4.02)	**<0.001**	1 (ref.)	-0.61 (-2.68, 1.46)	-2.27 (-4.55, 0.02)	**-7.94 (-10.60, -5.27)**	**<0.001**
Cr	-1.90 (-2.84, -0.96)	**<0.001**	1 (ref.)	**-3.32 (-5.60, -1.04)**	**-4.03 (-6.70, -1.36)**	**-6.62 (-9.42, -3.81)**	**<0.001**
Mn	-1.73 (-2.70, -0.75)	**0.001**	1 (ref.)	-0.60 (-3.17, 1.96)	-0.21 (-2.62, 2.21)	**-4.18 (-6.85, -1.50)**	**0.005**
Co	-2.97 (-4.83, -1.11)	**0.002**	1 (ref.)	**-2.16 (-4.30, -0.03)**	**-4.30 (-6.59, -2.00)**	**-7.02 (-9.46, -4.59)**	**<0.001**
Ni	-1.27 (-2.26, -0.29)	**0.011**	1 (ref.)	-1.82 (-4.04, 0.40)	**-2.70 (-5.14, -0.26)**	**-4.04 (-6.66, -1.42)**	**0.002**
Cu	-5.67 (-8.10, -3.23)	**<0.001**	1 (ref.)	-0.82 (-3.24, 1.60)	**-3.55 (-6.32, -0.78)**	**-8.33 (-11.62, -5.04)**	**<0.001**
Zn	-5.52 (-7.21, -3.83)	**<0.001**	1 (ref.)	-0.77 (-2.97, 1.43)	**-4.21 (-6.73, -1.69)**	**-7.08 (-9.99, -4.17)**	**<0.001**
As	-0.86 (-1.77, 0.04)	0.062	1 (ref.)	-1.94 (-4.26, 0.37)	**-4.00 (-6.51, -1.50)**	**-2.89 (-5.19, -0.60)**	**0.005**
Se	-10.79 (-13.88, -7.70)	**<0.001**	1 (ref.)	-0.31 (-2.40, 1.78)	**-5.82 (-8.31, -3.33)**	**-9.26 (-11.77, -6.75)**	**<0.001**
Mo	-4.78 (-6.41, -3.15)	**<0.001**	1 (ref.)	-1.34 (-3.75, 1.08)	**-3.19 (-5.65, -0.73)**	**-6.43 (-8.99, -3.86)**	**<0.001**
Pd	-1.08 (-2.21, 0.05)	0.062	1 (ref.)	-1.38 (-3.62, 0.87)	-1.78 (-4.40, 0.83)	-1.49 (-4.07, 1.09)	0.266
Ag	-0.76 (-1.42, -0.10)	**0.024**	1 (ref.)	-0.74 (-3.03, 1.55)	**-2.78 (-5.48, -0.07)**	-2.12 (-4.65, 0.40)	**0.048**
Cd	-3.16 (-4.72, -1.60)	**<0.001**	1 (ref.)	-0.55 (-3.00, 1.88)	**-2.56 (-4.93, -0.20)**	**-3.09 (-5.83, -0.34)**	**0.012**
Sn	-1.42 (-2.40, -0.44)	**0.005**	1 (ref.)	-2.10 (-4.93, 0.73)	**-3.24 (-6.36, -0.12)**	**-4.60 (-7.78, -1.43)**	**0.004**
Sb	-0.92 (-1.66, -0.19)	**0.014**	1 (ref.)	-1.01 (-3.72, 1.70)	**-4.67 (-7.82, -1.51)**	**-6.67 (-10.55, -2.80)**	**<0.001**
I	-1.63 (-2.65, -0.60)	**0.002**	1 (ref.)	-1.08 (-3.39, 1.23)	**-3.47 (-6.11, -0.83)**	**-3.79 (-6.50, -1.08)**	**0.002**
Pt	-1.42 (-2.69, -0.16)	**0.028**	1 (ref.)	-0.09 (-2.58, 2.39)	-2.03 (-4.59, 0.53)	**-4.12 (-7.04, -1.21)**	**0.002**
Hg	-1.32 (-2.69, 0.05)	0.059	1 (ref.)	-2.18 (-4.53, 0.17)	**-2.82 (-5.31, -0.32)**	-2.51 (-5.30, 0.28)	0.079
Tl	-7.04 (-9.30, -4.78)	**<0.001**	1 (ref.)	-1.54 (-3.76, 0.68)	**-3.09 (-5.51, -0.67)**	**-7.72 (-10.40, -5.03)**	**<0.001**
Pb	-1.38 (-2.47, -0.29)	**0.013**	1 (ref.)	-0.56 (-3.00, 1.88)	-1.44 (-4.01, 1.12)	-2.35 (-4.94, 0.24)	0.056
Bi	-0.86 (-1.64, -0.08)	**0.031**	1 (ref.)	-0.18 (-2.71, 2.34)	-2.98 (-6.18, 0.21)	**-3.74 (-6.92, -0.56)**	**0.009**

Results are presented as β coefficients and 95% CI from multivariable linear regression models adjusted for age, sex, education level, marital status, smoking status, alcohol consumption, obesity, hypertension, diabetes, vitamin D, C-reactive protein, physical activity, study center. Robust standard errors clustered by family code were used. Continuous associations were assessed using log-transformed fractional excretion (FE) levels. Quartile-based analyses were performed with the lowest quartile (Q1) as the reference group. P for trend was calculated by treating quartile categories as an ordinal variable in linear regression models. Abbreviations: eGFR, estimated glomerular filtration rate; FE, fractional excretion; CI, confidence interval.

Supplementary Table S9: Associations of fractional excretion of individual elements with CKD. SKIPOGH study, Lausanne, Switzerland.

Elements	Continuous		Quartiles				
	OR (95% CI)	*P* value	Q1	Q2 OR (95% CI)	Q3 OR (95% CI)	Q4 OR (95% CI)	*P* for trend
Li	1.08 (0.839,1.380)	0.563	1 (ref.)	1.24 (0.49,3.14)	1.44 (0.57,3.65)	1.14 (0.43,3.03)	0.728
Be	1.13 (0.939,1.367)	0.191	1 (ref.)	1.75 (0.69,4.47)	1.21 (0.50,2.92)	1.69 (0.69,4.10)	0.419
Al	1.31 (0.990,1.721)	0.059	1 (ref.)	1.66 (0.61,4.54)	0.94 (0.35,2.57)	1.76 (0.64,4.83)	0.428
V	2.36 (1.103,5.038)	**0.027**	1 (ref.)	0.89 (0.28,2.82)	0.74 (0.26,2.12)	1.79 (0.71,4.50)	0.169
Cr	1.23 (0.892,1.710)	0.204	1 (ref.)	0.66 (0.23,1.89)	0.94 (0.28,3.15)	2.06 (0.64,6.70)	0.193
Mn	1.30 (0.873,1.934)	0.196	1 (ref.)	0.96 (0.30,3.03)	1.04 (0.36,3.03)	1.88 (0.68,5.20)	0.167
Co	1.30 (0.748,2.273)	0.350	1 (ref.)	0.44 (0.14,1.37)	0.69 (0.25,1.88)	1.16 (0.47,2.83)	0.384
Ni	0.94 (0.701,1.251)	0.658	1 (ref.)	0.95 (0.37,2.45)	1.04 (0.40,2.69)	1.16 (0.46,2.92)	0.678
Cu	2.57 (1.525,4.344)	**<0.001**	1 (ref.)	1.50 (0.44,5.13)	1.04 (0.32,3.36)	**5.13 (1.64,16.02)**	**0.002**
Zn	3.91 (1.884,8.128)	**<0.001**	1 (ref.)	0.67 (0.18,2.48)	0.72 (0.23,2.27)	**3.13 (0.99,9.91)**	**0.023**
As	1.06 (0.762,1.486)	0.716	1 (ref.)	0.75 (0.29,1.93)	1.59 (0.67,3.79)	1.34 (0.55,3.25)	0.251
Se	1.57 (0.705,3.490)	0.270	1 (ref.)	0.88 (0.34,2.25)	0.54 (0.17,1.68)	1.50 (0.60,3.72)	0.451
Mo	0.78 (0.433,1.401)	0.404	1 (ref.)	0.67 (0.29,1.58)	0.68 (0.30,1.55)	0.83 (0.37,1.90)	0.677
Pd	0.70 (0.473,1.022)	0.065	1 (ref.)	0.51 (0.22,1.19)	0.87 (0.38,2.02)	0.39 (0.14,1.10)	0.160
Ag	1.24 (0.995,1.556)	0.056	1 (ref.)	1.15 (0.41,3.20)	1.63 (0.64,4.16)	2.10 (0.86,5.16)	0.079
Cd	0.99 (0.631,1.565)	0.977	1 (ref.)	1.80 (0.72,4.53)	1.19 (0.44,3.27)	1.11 (0.43,2.87)	0.678
Sn	1.20 (0.895,1.601)	0.226	1 (ref.)	1.80 (0.80,4.05)	0.82 (0.29,2.30)	1.87 (0.65,5.36)	0.543
Sb	1.16 (0.868,1.559)	0.310	1 (ref.)	1.72 (0.75,3.97)	1.29 (0.48,3.49)	3.80 (0.47,30.81)	0.308
I	0.91 (0.583,1.422)	0.679	1 (ref.)	0.73 (0.29,1.84)	0.48 (0.17,1.37)	1.06 (0.41,2.75)	0.782
Pt	1.17 (0.835,1.636)	0.363	1 (ref.)	1.96 (0.51,7.59)	1.65 (0.38,7.26)	1.67 (0.44,6.34)	0.610
Hg	0.81 (0.517,1.270)	0.359	1 (ref.)	0.94 (0.37,2.40)	0.70 (0.25,1.92)	0.63 (0.23,1.72)	0.293
Tl	1.90 (0.727,4.952)	0.191	1 (ref.)	0.40 (0.13,1.28)	0.65 (0.23,1.81)	1.20 (0.47,3.09)	0.366
Pb	1.16 (0.831,1.610)	0.387	1 (ref.)	2.44 (0.90,6.61)	1.67 (0.60,4.64)	1.69 (0.70,4.10)	0.611
Bi	1.17 (0.950,1.431)	0.142	1 (ref.)	0.90 (0.30,2.68)	1.73 (0.53,5.63)	1.72 (0.51,5.76)	0.171

Results are expressed as odds ratios (OR) with 95% CI. Logistic regression models were adjusted for age, sex, education level, marital status, smoking, alcohol consumption, physical activity, obesity, hypertension, diabetes, vitamin D, and C-reactive protein, study center. Robust standard errors clustered by family code were used. The continuous model represents the association per one-unit increase in log-transformed fractional excretion (FE). Quartile models compare each quartile (Q2–Q4) to the reference (Q1), and p for trend was calculated by modeling the median value of each quartile as a continuous variable. Abbreviations: CKD, chronic kidney disease; FE, fractional excretion; CI, confidence interval.

Supplementary Figure S1: Conceptual directed acyclic graph (DAG) for covariate selection in the analysis of 24-hour urinary element excretion and kidney function.

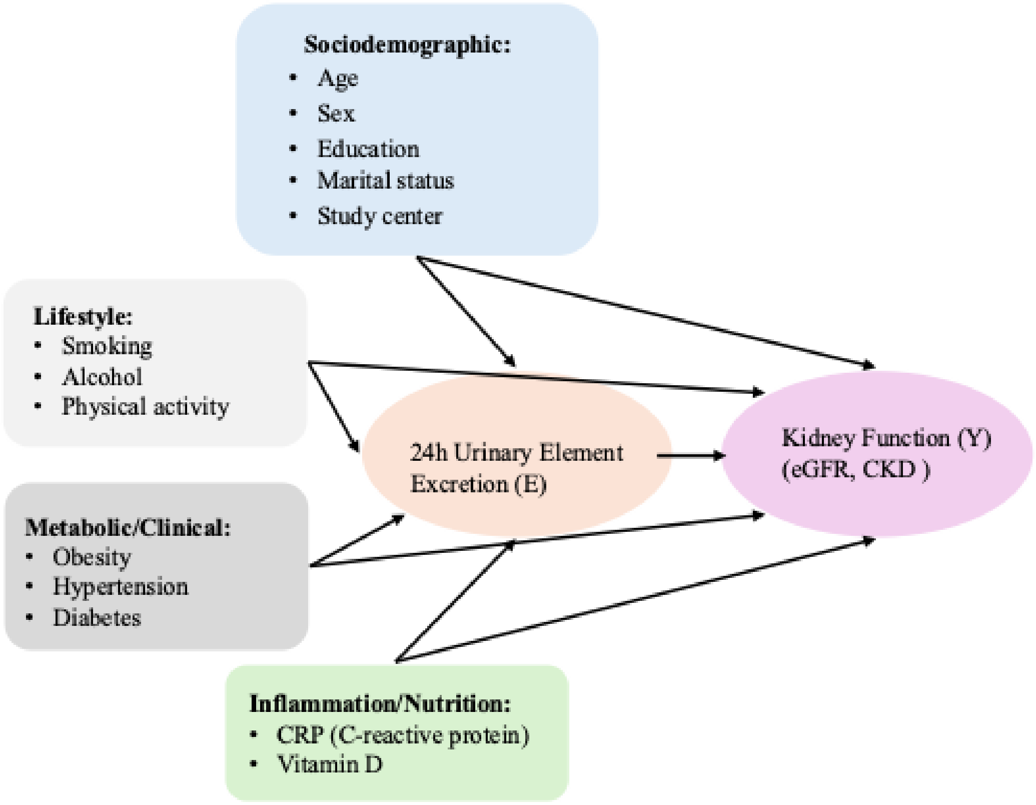

Directed acyclic graph (DAG) illustrating the assumed relationships between 24-hour urinary element excretion (E) and kidney function outcomes (Y: eGFR and CKD). Sociodemographic characteristics, lifestyle behaviors, metabolic/clinical factors, and nutritional/inflammatory biomarkers were considered potential common causes of exposure and outcome and were therefore included as adjustment variables. The DAG represents assumed relationships based on prior epidemiological evidence and biological plausibility.

Supplementary Figure S2: Conceptual framework illustrating the relationships between plasma element levels, kidney function (eGFR/CKD), and 24-hour urinary element excretion in a cross-sectional setting.

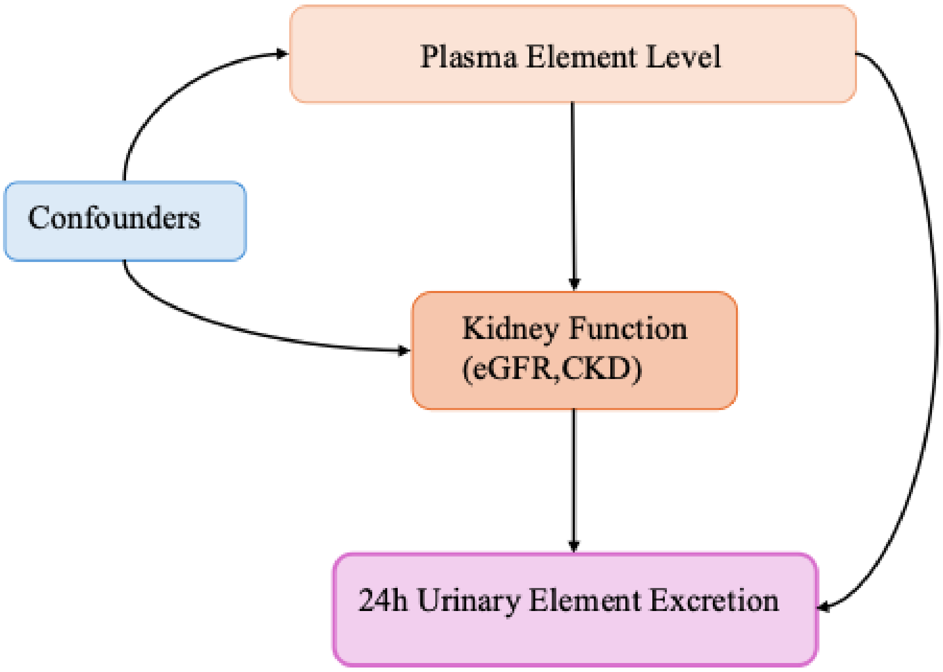

Plasma element levels were conceptualized as biomarkers of systemic exposure. In a cross-sectional setting, 24-hour urinary element excretion reflects both circulating element levels and kidney handling capacity. Sociodemographic, lifestyle, and clinical factors were considered potential common determinants of plasma element levels and kidney function. Arrows represent hypothesized associations in a cross-sectional setting and should not be interpreted as causal effects or temporal ordering. Relationships between plasma element levels and kidney function may be bidirectional. Because urinary excretion is influenced by glomerular filtration and tubular transport, reverse causation cannot be excluded. Abbreviations: eGFR, estimated glomerular filtration rate; CKD, chronic kidney disease.

Supplementary Figure S3: Selection of participants from SKIPOGH 2009–2013. SKIPOGH study, Lausanne, Switzerland.

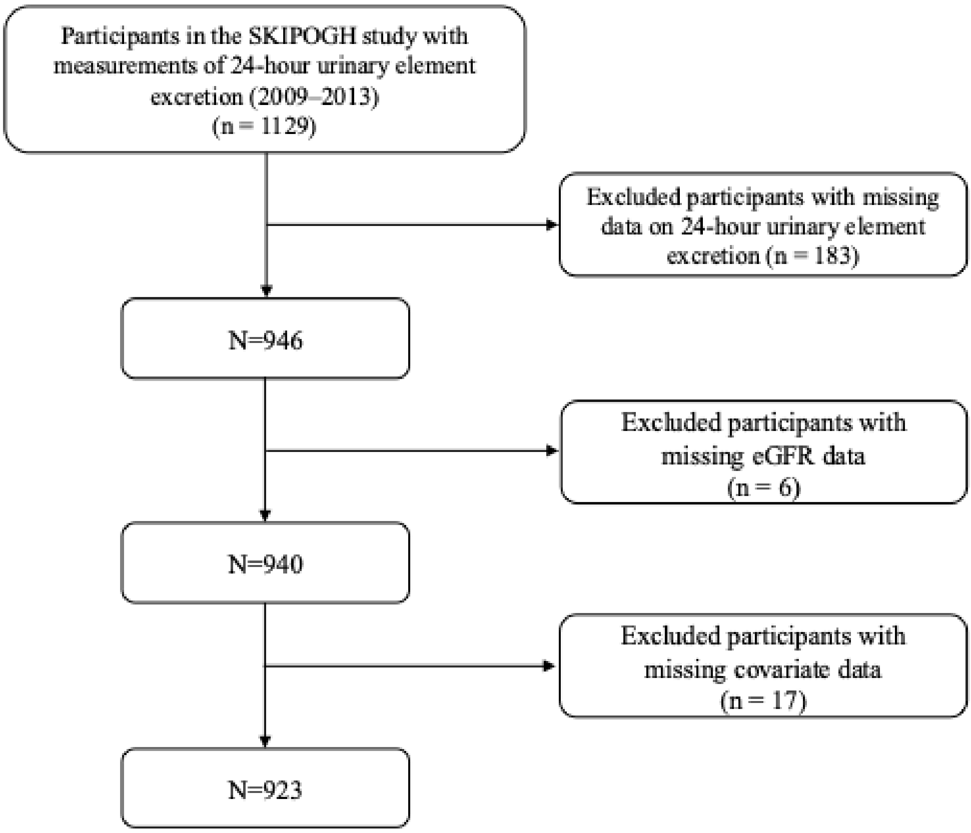

eGFR: estimated glomerular filtration rate.

Supplementary Figure S4: Non-linear associations between 24-hour urinary excretion of 24 elements and eGFR based on restricted cubic spline models. SKIPOGH study, Lausanne, Switzerland.

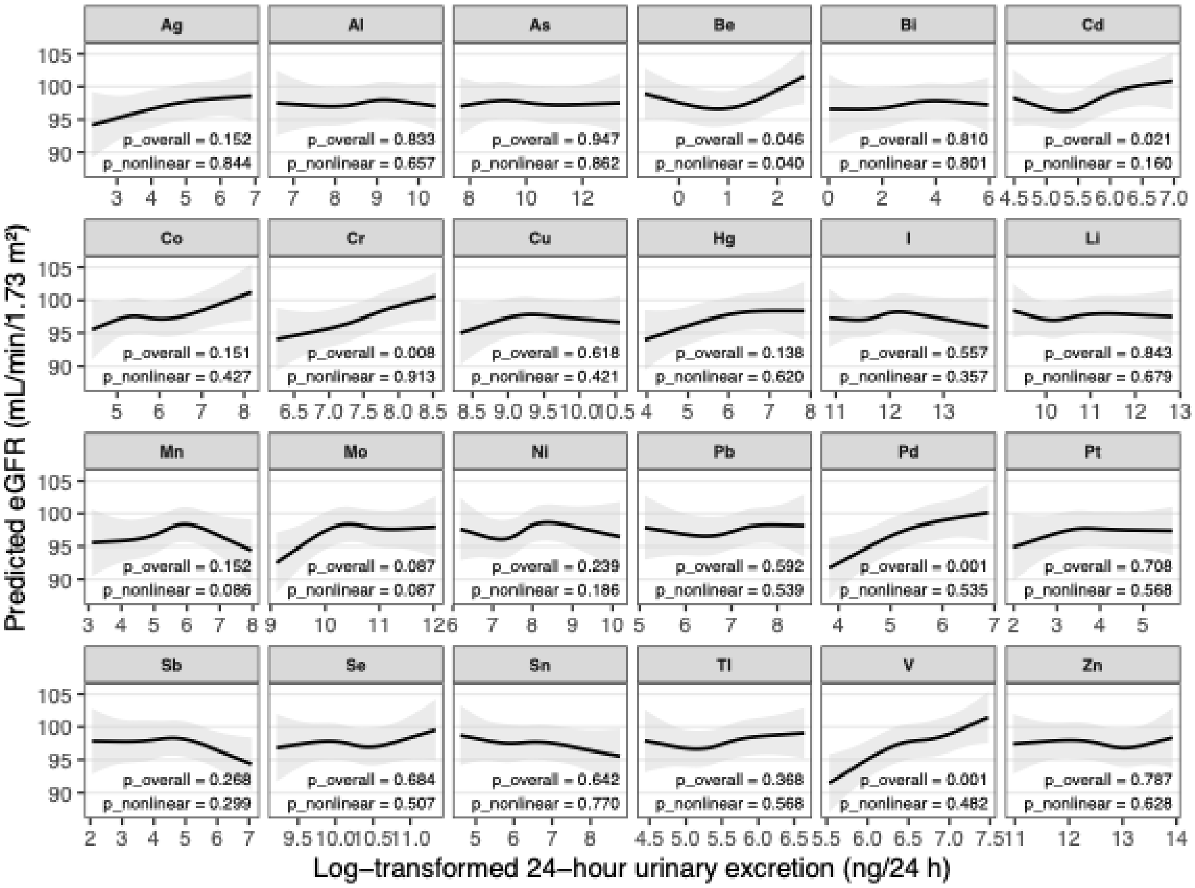

Restricted cubic spline (RCS) models were used to assess potential non-linear associations between log-transformed 24-hour urinary element excretion (ng/24 h) and predicted eGFR (mL/min/1.73 m²). Models were adjusted for age, sex, education level, marital status, smoking status, alcohol consumption, obesity, hypertension, diabetes, vitamin D, C-reactive protein, physical activity, and study center, with robust standard errors clustered by family code. Solid lines represent adjusted predictions, and shaded areas indicate 95% confidence intervals. P values for overall and non-linear associations are shown in each panel. Abbreviations: eGFR, estimated glomerular filtration rate.

Supplementary Figure S5: Non-linear associations between 24-hour urinary excretion of 24 elements and CKD based on restricted cubic spline models. SKIPOGH study, Lausanne, Switzerland.

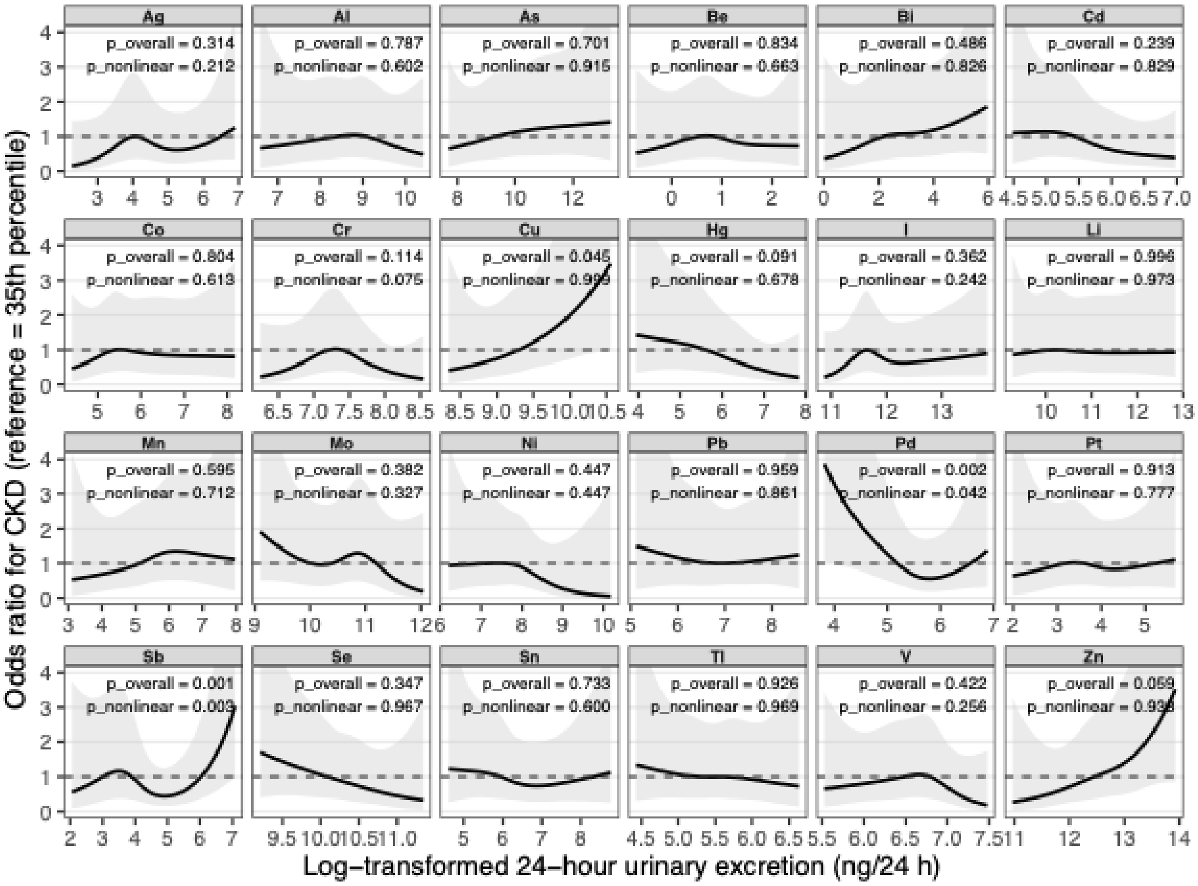

Restricted cubic spline (RCS) models were used to examine potential non-linear associations between log-transformed 24-hour urinary element excretion (ng/24 h) and odds of CKD. Models were adjusted for age, sex, education level, marital status, smoking status, alcohol consumption, obesity, hypertension, diabetes, vitamin D, C-reactive protein, physical activity, and study center, with robust standard errors clustered by family code. Solid lines represent adjusted odds ratios, and shaded areas indicate 95% confidence intervals. The dashed horizontal line indicates an odds ratio of 1. The 35th percentile was used as the reference value. P values for overall and non-linear associations are shown in each panel. Abbreviations: CKD, chronic kidney disease.

Supplementary Figure S6: Pearson correlation matrix of log-transformed 24-hour urinary excretion levels of 24 elements. SKIPOGH study, Lausanne, Switzerland.

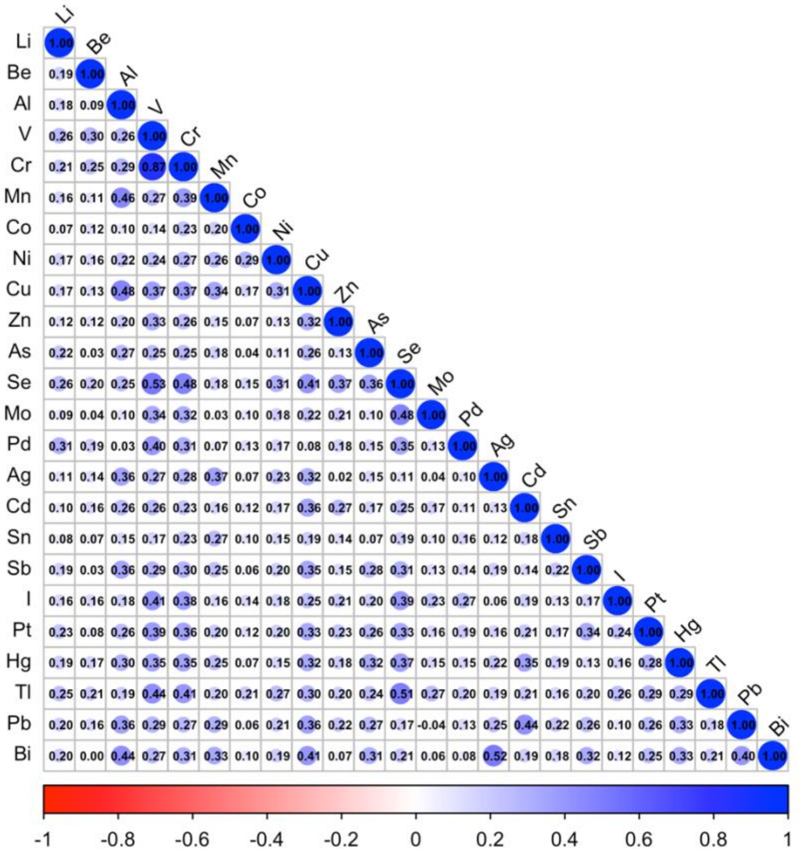

Pearson correlation matrix of log-transformed 24-hour urinary element excretion levels for 24 elements. Circle size and color intensity represent the magnitude of the Pearson correlation coefficient (r), ranging from −1 to 1. Only the lower triangle of the matrix is shown.
